# Meta-control of the exploration-exploitation dilemma emerges from probabilistic inference over a hierarchy of time scales

**DOI:** 10.3758/s13415-020-00837-x

**Published:** 2020-12-28

**Authors:** Dimitrije Marković, Thomas Goschke, Stefan J. Kiebel

**Affiliations:** 1 Chair of Neuroimaging, Faculty of Psychology, Technische Universität Dresden, 01062 Dresden, Germany; 2 Chair of General Psychology, Faculty of Psychology, Technische Universität Dresden, 01062 Dresden, Germany; 3 Centre for Tactile Internet with Human-in-the-Loop (CeTI), Technische Universität Dresden, 01062 Dresden, Germany

**Keywords:** Meta-control, Arbitration, Exploration-exploitation dilemma, Hierarchy of time scales, Active inference

## Abstract

Cognitive control is typically understood as a set of mechanisms that enable humans to reach goals that require integrating the consequences of actions over longer time scales. Importantly, using routine behaviour or making choices beneficial only at short time scales would prevent one from attaining these goals. During the past two decades, researchers have proposed various computational cognitive models that successfully account for behaviour related to cognitive control in a wide range of laboratory tasks. As humans operate in a dynamic and uncertain environment, making elaborate plans and integrating experience over multiple time scales is computationally expensive. Importantly, it remains poorly understood how uncertain consequences at different time scales are integrated into adaptive decisions. Here, we pursue the idea that cognitive control can be cast as active inference over a hierarchy of time scales, where inference, i.e., planning, at higher levels of the hierarchy controls inference at lower levels. We introduce the novel concept of meta-control states, which link higher-level beliefs with lower-level policy inference. Specifically, we conceptualize cognitive control as inference over these meta-control states, where solutions to cognitive control dilemmas emerge through surprisal minimisation at different hierarchy levels. We illustrate this concept using the exploration-exploitation dilemma based on a variant of a restless multi-armed bandit task. We demonstrate that beliefs about contexts and meta-control states at a higher level dynamically modulate the balance of exploration and exploitation at the lower level of a single action. Finally, we discuss the generalisation of this meta-control concept to other control dilemmas.

## Introduction

The concept of cognitive control is generally used as a summary term for a set of processes that enable humans to flexibly configure perceptual, emotional, and response selection processes in accordance with superordinate goals. These processes are especially pronounced when goal attainment requires novel or nonroutine action sequences, and there is competition from otherwise stronger habitual or impulsive responses (Botvinick and Cohen, 2014; Egner, 2017; Goschke, 2003; Goschke, 2013; Miller and Cohen, 2001). Cognitive control is considered essential for some of the most advanced cognitive capacities of humans, such as the ability to pursue long-term goals and to respond flexibly to changing contexts and task demands.

However, much of the experimental research on cognitive control has focused on relatively simple laboratory tasks, as, for instance, interference paradigms, such as Stroop or flanker task (Kalanthroff, Davelaar, Henik, Goldfarb and Usher, 2018; Scherbaum, Fischer, Dshemuchadse and Goschke, 2011), or paradigms assessing cognitive flexibility, such as task switching (Koch, Poljac, Muller and Kiesel, 2018). Many of these tasks are designed to induce conflicting internal representations, which trigger responses that are in contradiction to the instructed task goal and may lead to an incorrect response. Such tasks have been remarkably useful as psychological “probes” into component mechanisms of cognitive control, such as response inhibition or goal shielding, as they enable researchers to study how the brain copes with crosstalk between conflicting representations and competing responses. Accordingly, many computational models of cognitive control postulate a hierarchical mechanism (Botvinick, Niv and Barto, 2009), where higher-level representations of goals or task-sets serve as a biasing signal, which modulates processing at a lower level, such that information congruent with instructed goals gains higher priority in determining the selection of responses (Cohen, 2017; Goschke, 2003; Goschke, 2013; Miller and Cohen, 2001; Musslick, Jang, Shvartsman, Shenhav and Cohen, 2018; Scherbaum, Dshemuchadse, Ruge and Goschke, 2012). More recently, hierarchical models have been used to establish how the brain might determine the intensity and allocation of biasing signals to specific tasks, based on the estimated costs and benefits of recruitment of control (Shenhav, Botvinick and Cohen, 2013).

These approaches to study and model cognitive control focus on a specific class of cognitive control tasks that typically require only short-term planning within a single trial. This means that these tasks differ in a key aspect from real-life goal-reaching scenarios in which humans typically use cognitive control; i.e., to make an action in their everyday environment, humans must predict the consequences of this action over both short and long time periods and take into account the behaviour of other agents and relevant environmental dynamics. Clearly, predicting the future and planning one’s behaviour is a critical part of cognitive control, because it is essential for reaching desired goals. Consequently, in recent years, an increasing number of studies have investigated the role of planning over multiple future trials using sequential decision-making tasks (Economides, Guitart-Masip, Kurth-Nelson and Dolan, 2014; Kolling, Wittmann and Rushworth, 2014; Schwartenbeck, FitzGerald, Mathys, Dolan and Friston, 2015; Shenhav, Straccia, Musslick, Cohen and Botvinick, 2018).

### Cognitive control dilemmas and meta-control

While research in the past decades has substantially deepened insights into the computational mechanisms and neural systems that mediate our capacity for cognitive control, the meta-level processes that regulate complementary cognitive control processes itself remain poorly understood. Agents with an extended future time perspective, which pursue goal-directed action in changing and uncertain environments, are confronted with a set of antagonistic adaptive challenges. These challenges can be conceived of as fundamental *control dilemmas,* which require a context-sensitive adjustment of complementary control modes and control parameters (Goschke, 2003; Goschke, 2013; Goschke and Bolte, 2014). For instance, while the ability to shield long-term goals from competing responses promotes behavioural stability and persistence, it increases the risk of overlooking potentially significant changes in the environment and may lead to rigid and perseverative behaviour. Conversely, while a broad scope of attention supports background-monitoring for potentially significant changes and facilitates flexible goal switching, it also increases distractibility and may lead to volatile behaviour that is driven by every minor change in the environment (Dreisbach and Goschke, 2004; Goschke and Bolte, 2014). Agents must thus not only decide which action is best suited to attain a goal, but they have to cope with meta-control problems (e.g., should one ignore an unexpected change and shield a current goal from distraction or should one process task-irrelevant information, because it may signal that one should switch to a different goal?). Given that antagonistic adaptive constraints cannot be satisfied simultaneously to an arbitrary degree, because stable versus flexible control modes incur complementary costs and benefits, goal-directed agents must solve *meta-control problems.* This raises the questions how the brain achieves a context-sensitive balance between complementary control modes and how control parameters are adjusted to optimize goal attainment in changing and uncertain environments.

While control dilemmas arise in a range of processing domains (e.g., goal shielding vs. goal shifting; focused attention vs. background-monitoring; anticipation of future needs vs. responding to current desires; computationally demanding but flexible goal-directed control vs. less demanding but inflexible habitual control), we focused on the trade-off between exploration and exploitation as one of the most widely investigated control dilemmas (Addicott, Pearson, Sweitzer, Barack and Platt, 2017; Blanchard and Gershman, 2018; Cohen, McClure and Yu, 2007). It is obviously adaptive for agents to exploit and select those actions that maximized reward in the past. However, to learn about such actions or find better ones, agents must explore previously untried actions. Thus, exploitation may prevent learning about task-relevant actions and states; conversely, exploration may return relatively little reward or even lead to risky behaviour.

A widely used formulation of meta-control, i.e., of deciding how to decide (Boureau, Sokol-Hessner and Daw, 2015), is as a competition between automatic responses (e.g., habits) and elaborate choices (e.g., planned responses) where the opportunity cost of assigning limited computational resources to planned decisions is weighted against the possibility of improved outcomes in the future. In other words, meta-control between automatic and planned behaviour often is cast as a trade-off between gains and costs (Shenhav et al., 2013). Although this approach can link meta-control problems from different domains (e.g., shielding-shifting, selection-monitoring, or exploration-exploitation dilemmas (Goschke, 2013; Goschke and Bolte, 2014)), in its standard formulation, it does not account for the fact that the future is inherently uncertain and that different behavioural policies will have different effects on that uncertainty, i.e., lead to different information gain or loss (i.e., lead to different information gain). In practice and independent of the task, behaviour that leads to precise beliefs about possible action-outcome contingencies should be preferred to the one that results in imprecise beliefs about action-outcome contingencies, as the resulting estimates of expected rewards and costs become more reliable and less computationally demanding. Recent research on decision making under uncertainty has demonstrated the importance of taking into account information gain for understanding human choice behaviour (Dezza, Angela, Cleeremans and Alexander, 2017).

We will pursue a different approach and cast meta-control as an inference problem (Schwartenbeck et al., 2019). Meta-control will appear costly when one has only vague prior knowledge about which behavioural policy one should follow as the future outcomes of one’s behaviour become difficult to predict. For example, if one would know with absolute certainty that eating sugar, in any amount, will badly impact one’s health in the future, deciding not to eat a tasty cake would become a trivial choice. In reality, there is uncertainty on how consuming sugar impacts our future health; e.g., occasional consumption may not have negative consequences for one’s health. Given this intrinsic uncertainty, deciding about consuming sugar is an inference problem, where one’s beliefs about the relationship between consuming sugar and health will drive behaviour. Therefore, when making decisions, we can link the perceived “costs” and an apparent “loss of control” to uncertain beliefs about optimal behaviour, as a consequence of vague beliefs about future outcomes.

Hence, following the idea that both perception and action can be formulated as probabilistic (Bayesian) inference (Botvinick and Toussaint, 2012; Friston, 2010), we will approach the question of how meta-control is computed in an analogous fashion in terms of a single optimisation principle: minimisation of expected surprisal about future outcomes within a hierarchical architecture (Pezzulo, Rigoli and Friston, 2015), that is, as hierarchical active inference (Friston, 2010; Friston, Rosch, Parr, Price and Bowman, 2018; Pezzulo et al., 2015). In what follows, we will introduce basic concepts of hierarchical active inference and demonstrate the emergence of meta-control using the exploration-exploitation dilemma as an example. Briefly, hierarchical inference of external states (contexts), internal states (meta-control states), and control signals (actions) results in adaptive arbitration between exploratory and exploitative choices, where meta-control states at a higher level of the hierarchy constrain prior beliefs about available policies at the level below. The key point of this hierarchical model is that meta-control states encode an agent’s previously acquired beliefs how it should control its own behaviour in the current context.

Before we continue, we would like to acknowledge the vast literature on the exploration-exploitation dilemma in both machine learning (see e.g., Allesiardo, Féraud and Maillard, 2017; Houthooft et al., 2016; Schulz and Gershman, 2019) and cognitive neuroscience (see e.g., Addicott et al., 2017; Cohen et al., 2007; Daw, O'Doherty, Dayan, Seymour and Dolan, 2006; Geana, Wilson, Daw and Cohen, 2016; Laureiro-Martínez, Brusoni, Canessa and Zollo, 2015; Wilson, Geana, White, Ludvig and Cohen, 2014). The focus of previous theoretical research on the exploration-exploitation dilemma is closely linked to the so-called optimal stopping problem (Dubins, Savage, Sudderth and Gilat, 2014), in dynamic environments, e.g., a restless multi-armed bandit task (Liu, Liu and Zhao, 2012), where the exploration-exploitation dilemma corresponds to knowing when to stop sampling for information and switching to exploitation, and vice versa. The best known classical algorithms for resolving the dilemma are based either on upper confidence bounds to expected rewards (Auer, Cesa-Bianchi and Fischer, 2002; Garivier and Cappé, 2011) or Thompson sampling applicable to belief based (Bayesian) multi-armed bandits (Agrawal and Goyal, 2012).

The standard exploration-exploitation dilemma has also been described within the active inference framework (FitzGerald, Schwartenbeck, Moutoussis, Dolan and Friston, 2015; Friston et al., 2015), in which the balancing between exploration and exploitation is driven by minimisation of expected free energy (upper bound on expected surprisal) and resolved with local choices between rewarding and informative options. Interestingly, active inference based behavioural models are characterised both by random and directed exploration strategies (Schwartenbeck et al., 2019), similar to exploration strategies associated with human behaviour (Wilson et al., 2014). Complementary to these related works, we will show how hierarchical active inference can result in supressing or boosting the local exploration drive as a function of long-term predictions and goals. Therefore, we will focus on meta-control of behaviour where the agent can choose to be exploratory or exploitative given some long-term predictions and goals. We enabled this nonmyopic exploration by introducing temporally extended contexts at the higher level of the hierarchy. In this way, the agent can meta-control its behaviour depending on both the context it believes it is in, and as we show, the context it predicts to be in the future. Such a setup allows the agent to inhibit itself from exploring, despite knowing that exploration would lead to a significant gain of information about current context and other relevant hidden variables.

### Planning, uncertainty, and a hierarchy of time scales

Although not always obvious, human planning is for many tasks in daily life a computational feat yet unrivalled by any machine. Research in robotics and artificial intelligence has found that planning, in an online fashion, in our typically uncertain environment is a hard problem for artificial agents (Kurniawati, Du, Hsu and Lee, 2011). Even for mundane activities, such as safely driving a car through typical traffic, artificial planning performance is currently well below human routine performance (for a current review see Schwarting, Alonso-Mora and Rus, 2018). Although there are recent findings that artificial agents perform better than humans in specific planning tasks like playing the board game Go (Silver et al., 2017), the question is what makes planning challenging in scenarios, such as driving a car. We will focus on two of these features, which are also probably the most relevant for addressing cognitive control research questions.

First, for a goal-directed agent, most environments are packed with uncertainty. This uncertainty is induced by various sources (Soltani and Izquierdo, 2019), which make planning difficult because the number of possible ways in which the environment may develop grows massively the further into the future one tries to plan ahead. Second, in our environment, things change at different time scales, and we are additionally confronted with uncertainty about the relevance of different time scales and how representations at different time scales interact with each other. In other words, learning the relevant representations at different time scales for one’s planning and goal reaching is a problem in itself.

Recent experimental and theoretical research in the cognitive neurosciences demonstrated that these multiple time scales are a critical dimension along which the brain structures its environment (Badre and Nee, 2018; Chaudhuri, Knoblauch, Gariel, Kennedy and Wang, 2015; Dixon, Girn and Christoff, 2017; Kiebel, Daunizeau and Friston, 2008; Koechlin, Ody and Kouneiher, 2003). In the domain of cognitive control, the relevance of different time scales is well established in the context of, for instance, intertemporal choice conflicts, where agents have to choose between a smaller reward that can be obtained immediately versus a larger reward that can be obtained only after a delay (Dai, Pleskac and Pachur, 2018; Kable, 2014; Scherbaum, Dshemuchadse, Leiberg and Goschke, 2013). Hence, the conceptual backbone of the model that we describe below is that the representation of environmental dynamics is organized as a hierarchy of time scales (Kiebel et al., 2008). Similar modelling approaches have been proposed in cognitive control in the context of hierarchical reinforcement learning (HRL) (Botvinick and Weinstein, 2014; Holroyd and McClure, 2015) and also are naturally an increasingly relevant topic in artificial intelligence research (Bacon and Precup, 2018; Le, Vien and Chung, 2018; Mnih et al., 2015; Pang et al., 2019). In general, HRL models are based on the idea that action sequences can be chunked and represented as a new temporally extended action, the so-called option (Barto and Mahadevan, 2003; Sutton, Precup and Singh, 1999). For example, making tea lasts approximately 30 seconds and requires performing a series of actions. Each of these actions (e.g., to get some water) is at a faster, more fine-grained time scale and last only a few seconds. This principled idea to represent behaviour as a hierarchy of sequences also has been proposed as a way of interpreting recent findings in fields, such as speech (Hasson, Yang, Vallines, Heeger and Rubin, 2008), memory, and the hippocampus (Collin, Milivojevic and Doeller, 2017) and decision making (Hunt and Hayden, 2017).

In the psychology literature, the idea that goal-directed control is organised as a hierarchy with elements represented at different time scales can be traced back to concepts outlined for example by Miller, Galanter and Pribram (1960) and pursued in action control theories (Gollwitzer and Bargh, 1996; Heckhausen and Kuhl, 1985; Kuhl and Goschke, 1994). We will use the principle as exemplified by recent HRL modelling work but critically complement the resulting model by three components, which we believe are important to explain specific meta-control phenomena. Note that all three components have been used before in probabilistic modelling approaches and are not novel by themselves. Our point is that the combination of these specific model components enables an agent to learn how to balance its explorative and exploitative tendencies in a context-dependent fashion.

First, as motivated above, planning in our environment must incorporate various sources of uncertainty, which requires that we formulate the hierarchical model probabilistically (see *Methods* for details). Second, hierarchical reinforcement learning models previously applied in the cognitive neurosciences (Holroyd and McClure, 2015) typically assume that agents aim at maximizing future return, the so-called instrumental value (IV). This approach works well for modelling and analysing experimental tasks, which require participants to reach goals in an already well-learned task environment. However, when considering cases in which an agent has not yet learned its task environment, actions should not only serve the maximization of reward but also the reduction of uncertainty about task-relevant states and parameters (Ghavamzadeh, Pineau and Tamar, 2015). To be able to model such uncertainty-reducing, explorative actions of an agent, we will use the expected free energy, which combines instrumental value with the epistemic value of different actions, thereby leading to a reduction of uncertainty about the state of the world (Kaplan and Friston, 2018). Third and most importantly, we introduce specific hidden states: the meta-control states. Meta-control states constrain the prior over behavioural policies on the level below and do not encode environmental states but rather behavioural modes of the lower level.

In what follows, we will introduce active inference and its extension to hierarchical generative models, deep active inference. Importantly, beliefs about meta-control states will be entrained by the beliefs about the current context and the agent’s preferences to successfully perform the task. These beliefs about meta-control states limit behavioural policies to action sequences that are most likely to lead to a goal.

### Active inference

To set the scene for the proposed model, we briefly introduce the active inference framework, which is an application of the free-energy principle (Friston, 2010) to a sequential decision making under uncertainty, that is, a partially observable Markov decision process (POMDP) (Kaelbling, Littman and Cassandra, 1998; Littman, 2009). Importantly, belief states in active inference cover both beliefs about states and beliefs about policies. In other words, in active inference planning is also cast as an inference problem (Botvinick and Toussaint, 2012), with an imperative to minimises surprise about future outcomes, that is, the upper bound on surprise: the expected free energy.

Formally, we can express the expected free energy of a specific behavioural policy *π* at some future time step *τ* as (Schwartenbeck et al., 2019).

1where $\overset{\sim }{Q}=Q\left({o}_{\tau },{s}_{\tau },A|\pi \right)=P\left({o}_{\tau }|{s}_{\tau },A\right)Q\left({s}_{\tau }|\pi \right)Q(A)$  denotes a joint distribution over current beliefs about likelihoods *Q*(*A*) (i.e., state-outcome contingencies; $P\left({o}_t=i|{s}_t=j,A\right)={A}_{i,j}$, and beliefs $P\left({o}_{\tau }|{s}_{\tau },A\right)Q\left({s}_{\tau }|\pi \right)$ about states *s*_*τ*_ and outcomes *o*_*τ*_ at future step *τ* conditioned on a specific policy *π*. In the equation above, the epistemic value (EV) term corresponds to the expected information gain and the instrumental value (IV) term to the expected extrinsic reward, as *P*(*o*_*τ*_) encodes prior preferences over different outcomes *o*_*τ*_. Importantly, the prior preferences over outcomes do not define how likely different future outcomes are, but rather an intrinsic incentive that the agent will follow policies that lead to preferred outcomes. Flat prior preferences would lead to purely exploratory behaviour (either as random or directed exploration), whereas sharp prior preferences (centred over preferred outcomes) lead to exploitative behaviour. In practice, modulations of prior preferences results in changing the balance between exploratory and exploitative behavioural policies. In this work, we will introduce a hierarchical architecture that enables an agent to infer its own modulation of prior preferences to guide behaviour through different contexts.

In the context of active inference, a behavioural policy *π* is defined as a specific sequence of actions or control signals *u*; hence we write *π* = (*u*_*t*_, …, *u*_*T*_). This formulation is closely related to options, that is, the notation of temporally extended actions commonly used in reinforcement learning (Bacon, Harb and Precup, 2017), which also include more sophisticated high-level actions than fixed action sequences. To minimise the expected free energy, an agent should select those policies which it expects to lead to minimal surprisal, which corresponds to the following policy prior

2 $$ p\left(\pi \right)=\frac{1}{Z}{e}^{-\gamma {\sum}_{\tau =t}^TG\left(\pi, \tau \right)} $$

where *γ* denotes a free model parameter shaping prior precision, and *Z* the normalisation constant. Note that minimising free energy naturally leads to a lower choice uncertainty with decreased uncertainty about hidden states of the environment, as the significance of different behavioural policy becomes more evident.

Finally, we can express the full generative model, which defines the known relations between actions, states transitions, and outcomes, as

3 $$ p\left({O}_T,{S}_T,A,\pi \right)=p\left(\pi \right)p(A)\prod \limits_{t=1}^Tp\left({o}_t|{s}_t,A\right)p\left({s}_t|{s}_{t-1},\pi \right) $$

where *O*_*T*_ = (*o*_1_, …, *o*_*T*_), *S*_*T*_ = (*s*_1_, …, *s*_*T*_). Besides the prior over policies, the generative model is characterised by the prior over likelihoods p(A) (e.g., a Dirichlet distribution), the observation likelihood *p*(*o*_*t*_| *s*_*t*_, *A*), and the state transition probabilities *p*(*s*_*t*_|*s*_*t* − 1_, *π*). As policy *π* denotes a specific sequence of control states (*u*_1_, …, *u*_*T*_), the state transition probability at time step *t* is parametrised by the corresponding control signal *u*_*t*_, hence *p*(*s*_*t*_|*s*_*t* − 1_, *π*) ≡ *p*(*s*_*t*_| *s*_*t* − 1_, *u*_*t*_). Here, we will assume that at initial time step *t* = 1, the state *s*_1_ is completely defined by the control states, hence *p*(*s*_1_| *s*_0_, *π*) = *p*(*s*_1_| *π*).

We define the inversion of the generative model (eq. (3)), that is, inference of hidden states and policies from outcomes, as a process of minimising variational free energy *F*, with respect to approximate posterior beliefs over hidden variables *Q*(*A*, *S*_*T*_, *π*). Hence, we obtain the belief update equations from an approximate inference scheme, the variational inference. We describe the formal definition of the variational free energy and the details of the belief update equations for the hierarchical variant of the generative model in the *Methods* section.

### Deep temporal models

We will refer to the extension of active inference to hierarchical generative models with implicit temporal structure (deep temporal models) as deep active inference (Friston et al., 2018). Deep temporal models are defined as hierarchical generative models with increasing levels of hierarchy capturing slower time scales. Furthermore, different level of the hierarchy are connected such that auxiliary outcomes at an upper level of the hierarchy modulate prior beliefs at the lower level of the hierarchy. This link between neighbouring hierarchical levels allows formulating message passing algorithms between adjacent levels akin to the message passing algorithms used for sequential inference within a single level of the hierarchy.

As our goal is to describe how the cognitive control and the resolution of control dilemmas naturally emerge within deep active inference, we denote as the meta-control state a state, at an upper level of the hierarchy, which imposes (via link probabilities) constraints on prior beliefs (e.g., about policies, states, or state-transitions) at the adjacent lower level. Importantly, transitions between different realisations of meta-control states are mediated with hierarchy specific control signals, where a sequence of control signals corresponds to a policy at a given level of the hierarchy. The model inversion and formation of beliefs about hidden states, policies, and meta-control states at an upper level of the hierarchy entrain the sequential inference process on the level below. Finally, the control signals at the lowest level of the hierarchy are mapped to choices (actions).

**Fig. 1:**
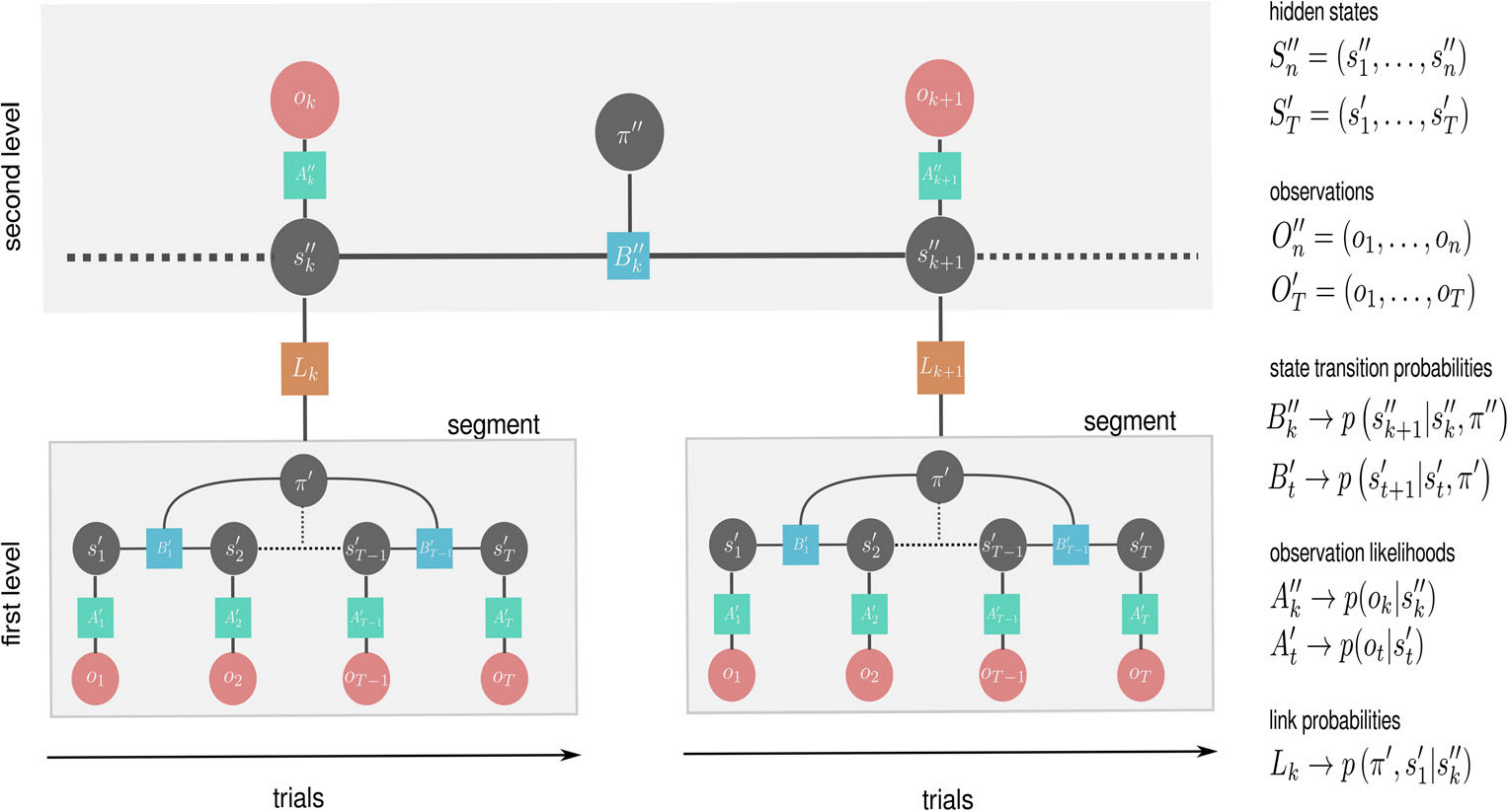
Factor graph representation of the hierarchical generative model for the presented task. The graph consists of two types of nodes: (i) Random variables (circles), which can be either evidence variables (red) whose value is observed or hidden state variables (grey) whose value has to be inferred. (ii) Factors (squares) that define the relationship between random variables. At the upper level of the hierarchy, the agent entertains beliefs (a probability distribution over the set of possible states) about the current context, context duration, and its meta-control state; hence, ${s}_k^{\prime \prime }=\left({c}_k^{\prime \prime },{d}_k^{\prime \prime },{i}_k^{\operatorname{}\prime \prime}\right)$, the c-i pair defines the observation likelihood of the outcome *o*_*k*_ at the end of a segment (success or failure). The duration variable *d* is not linked to observations but rather modulates the context transition probability, defining the moment of context transition. The behavioural policy at the second level of the hierarchy ${\pi}^{\prime \prime }$ selects the appropriate meta-control state for the next segment. The link probability *L*_*k*_ relates second level states to the prior beliefs about the lower level states ${s}_0^{\prime }$ and policies ${\pi}^{\prime }$. The lower level states factorise into the chosen options $\left({l}_0^{\prime },\dots, {l}_T^{\prime}\right)$ and auxiliary context and meta-control states ${c}_k^{\prime },{i}_k^{\prime }$ (fixed states during each segment, hence ${c}_0^{\prime }=\dots ={c}_T^{\prime}\equiv {c}_k^{\prime }$, and ${i}_0^{\prime }=\dots ={i}_T^{\prime}\equiv {i}_k^{\prime }$) which capture lower level information about upper level states. Importantly, the auxiliary context states ${c}_k^{\prime }$ determine currently active observation likelihood, and the auxiliary control states ${i}_k^{\prime }$ set prior over policies $p\left({\pi}^{\prime }|{i}_k^{\prime}\right)$ at the first level of the hierarchy. For details see the *Methods* section.

We will limit the deep temporal model to a two-level hierarchy which we will use for simulating behaviour in a toy example below. The two-level deep temporal model presented in Fig. 1 can be expressed with the following joint probability distributions at different levels of the hierarchy

- *Lower level*

4 $$ \overline{p}\left({O}_T^{\prime },{S}_T^{\prime },{A}^{\prime },{\pi}^{\prime }|{o}_k^{\prime \prime}\right)=\overline{p}\left({A}^{\prime}\right)p\left({o}_1^{\prime}\right|{s}_1^{\prime },{A}^{\prime}\left)p\left({\pi}^{\prime },{s}_1^{\prime }|{s}_k^{\prime \prime}\right)\prod \limits_{t=2}^Tp\left({o}_t^{\prime}\right|{s}_t^{\prime },{A}^{\prime}\right)p\left({s}_t^{\prime}\right|{s}_{t-1}^{\prime },{\pi}^{\prime}\Big) $$

Where ${s}_t^{\prime }$ denotes hidden states at the lower level at trial *t*, ${s}_k^{\prime \prime }$ hidden states at the upper level during the current *k*th segment. As before, ${o}_t^{\prime }$ denotes an outcome, ${\pi}^{\prime }$ behavioural policy and ${A}^{\prime }$ the emission probability matrix which defines likelihood of outcomes ${o}_t^{\prime }$ in different states ${s}_t^{\prime }$. We use the bar notation in $\overline{p}\left({A}^{\prime}\right)$ to denote the dependence on the experience in past segments, hence $\overline{p}\left({A}^{\prime}\right)=p\left({A}^{\prime }|\ {\left[{O}_T^{\prime}\right]}^{1:k-1}\right)$, where ${\left[{O}_T^{\prime}\right]}^{1:k-1}=\left({\left[{o}_1^{\prime },\dots, {o}_T^{\prime}\right]}^1,\dots, {\left[{o}_1^{\prime },\dots, {o}_T^{\prime}\right]}^{k-1}\right)$ denotes the sequence of observed outcomes.

- *Upper level*

5 $$ \overline{p}\left({o}_k^{\prime \prime },{s}_k^{\prime \prime },{A}^{\prime \prime },{\pi}^{\prime \prime}\right)=\overline{p}\left({A}^{\prime \prime}\right)p\left({\pi}^{\prime \prime}\right)\overline{p}\left({s}_k^{\prime \prime }|{\pi}^{\prime \prime}\right)p\left({o}_k^{\prime \prime }|{s}_k^{\prime \prime },{A}^{\prime \prime}\right) $$

where $\overline{p}\left({A}^{\prime \prime}\right)=p\left({A}^{\prime \prime }|{O}_{k-1}^{\prime \prime}\right)$ corresponds to an approximate posterior estimate of state outcome mappings at the end of the previous (*k* − 1)th segment. Similarly, $\overline{p}\left({s}_k^{\prime \prime }|{\pi}^{\prime \prime}\right)={\sum}_{s_{k-1}^{\prime \prime }}p\left({s}_k^{\prime \prime }|{s}_{k-1}^{\prime \prime },{\pi}^{\prime \prime}\right)p\left({s}_{k-1}^{\prime \prime }|{O}_{k-1}^{\prime \prime}\right)$, denotes the predictive probability over the hidden states ${s}_k^{\prime \prime }$ in the current segment.

Note that the full generative model is obtained by multiplying conditional joint distributions at different levels of the hierarchy. Hence, the complete generative distribution is defined as

6 $$ \overline{p}\left({O}_T^{\prime },{S}_T^{\prime },{A}^{\prime },{\pi}^{\prime },{o}_k^{\prime \prime },{s}_k^{\prime \prime },{A}^{\prime \prime },{\pi}^{\prime \prime}\right)=\overline{p}\left({O}_T^{\prime },{S}_T^{\prime },{A}^{\prime },{\pi}^{\prime }|{s}_k^{\prime \prime}\right)\overline{p}\left({o}_k^{\prime \prime },{s}_k^{\prime \prime },{A}^{\prime \prime },{\pi}^{\prime \prime}\right) $$

We provide the details on the corresponding approximate inference scheme and fixed model parameters in the Methods section. In what follows we will introduce the experimental task (the generative process) and the corresponding behavioural model.

## Results

### Toy example

To illustrate emergent meta-control we will use a sequential decision making task, similar to behavioural tasks in which participants have to collect points in a series of trials to surpass a known point threshold (Cuevas Rivera, Ott, Marković, Strobel and Kiebel, 2018; Kolling et al., 2014). The task can be considered a generalization of dynamic multi-armed bandits (Gupta, Granmo and Agrawala, 2011) and consequently an extension to a probabilistic reversal learning task (Izquierdo, Brigman, Radke, Rudebeck and Holmes, 2017; Marković, Reiter and Kiebel, 2019). The goal of the task design is to create situations (contexts) in which exploration is either beneficial or detrimental to goal-reaching performance. Hence, an adaptive agent would be incentivised to supress or boost exploration depending on the hidden context. The task of this adaptive agent will be to learn useful behaviour for each context so that on future exposures to already experienced contexts, the agent can quickly adapt its behaviour to be more exploitative, or if necessary, exploratory. This makes the task more complex than other multi-armed bandit task used previously in exploration-exploitation research (Daw et al., 2006; Laureiro-Martínez et al., 2015; Schwartenbeck et al., 2019; Speekenbrink and Konstantinidis, 2015).

**Fig. 2:**
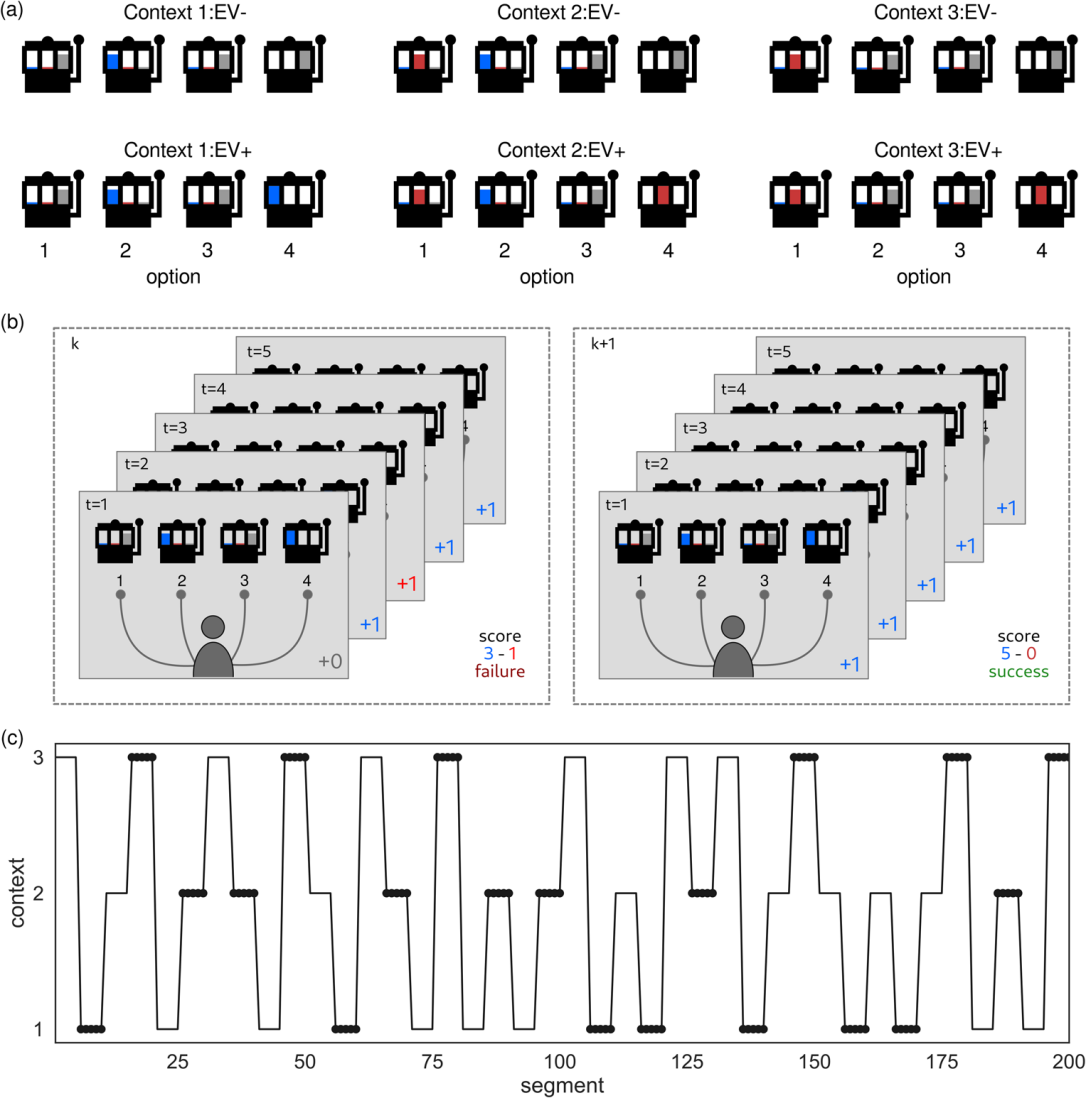
Illustration of the task structure. **a** Each context is defined by the coloured point probabilities associated with four different options. In the illustration the height of the coloured bar corresponds to the probability of obtaining a point of the corresponding colour (or no point denoted by a grey bar). Possible point probabilities are 0, 0.1, 0.8, and 1. In context variants EV*−* (top row), agents maximising only instrumental value will on average be more successful in reaching the segment-wise goal (surpass the threshold of four points of a single colour) than agents balancing both instrumental value and epistemic value. The opposite behaviour will be on average more successful for context variants EV+ (bottom row). Furthermore, the only difference between each context pair, e.g., contexts 1:EV*−* and 1:EV+, is the option which returns points with certainty, which corresponds to option 4 in each context. All other options return a blue, a red point or no point with 0.8 probability and the other two outcomes with 0.1 probability. Note that option types (point probabilities associated with an option) are shared across context, e.g., the point probabilities (0.8 blue point, 0.1 red point, 0.1 no point) are used four times in contexts 1:EV*−*, 1:EV+, 2:EV*−*, and 2:EV+. If an agent does not know the current context variant (EV*−* or EV+), the expected return of choosing the fourth option is lower compared to options associated with 0.1-point probability, e.g., option 1 in context pair 3:EV*−* and 3:EV+. However, option 4, which returns a point (or no point) with certainty, is the most informative, because it resolves the uncertainty about the context variant EV*−* or EV+. **b** Trial level task structure in two consecutive segments *k* and *k* + 1. To succeed, an agent has to collect at least 4 points of a single colour within a segment. For illustration purposes, we have assumed that the agent had selected option 2 five times in the *k*th segment but failed to reach the threshold. In the next segment, the agent selected the option 4 five times and passed the threshold. The true point probabilities are not known to the agent but only visualised here for clarification. **c** Context dynamics across segments that was fixed across simulations. The presence of circles denotes segments under context variants EV*−*, and the absence segments under context variants EV+.

In the task, runs of five trials form a segment, during which the agent can collect points in each trial by choosing one of four different options. Each of these options returns probabilistically one blue point, one red point, or no point. The number of collected points is evaluated after the fifth trial, where the reward is only given if the agent succeeded to collect at least four points of the same colour (Fig. 2b). For example, 4 red points and 0 blue points are rewarded, while 3 red points and 1 blue point are not rewarded. Although this setup and the following task description may appear quite complex in relation to typical cognitive control tasks like the Stroop task, we found that this level of task complexity is required to illustrate clear behavioural differences between an agent that can adapt exploratory tendencies and the one that works only in exploitative mode. We will elaborate on this point in more detail once we introduce the task specifics.

The simulated experiment consists of a series of five-trial segments where a switch to a new context occurs only between segments, hence at a slower time scale (Figure 2c). A context determines the probabilities of different point outcomes associated with each of the four options. Context changes occur whenever five segments, i.e., 25 trials, have been completed. The number of trials within a segment and the frequency of context changes were selected to make the task difficult enough to illustrate between agent differences.

Importantly, both context and the changes are hidden (not explicitly indicated). Hence, the agent can infer the current context only from a sequence of choice outcomes. We defined six different contexts (Fig. 2a). In three of these contexts, taking into account expected information gain when selecting options leads to a higher success probability compared to only considering the expected value of choices (once the choice-outcome contingencies are learned). Therefore, these three contexts incentivise an agent to combine both instrumental and epistemic value for action selection. In the rest of the paper, we will refer to these three contexts as EV+, short for “epistemic value +.” In the remaining three contexts, to be most successful, an agent should suppress its exploration tendencies (ignore information gain), so we call these contexts EV−. This means that a goal-directed agent, which employs meta-control, should adapt explorative tendencies depending on the context by either suppressing or boosting them.

The six contexts come in three pairs. Each context pair, e.g., context 1:EV− and 1:EV+ (Fig. 2a), consists of a context variant EV- in which policy selection should be based only on the instrumental value, and the context variant EV+ in which policy selection should be based on both instrumental and epistemic value. For each of the three context pairs, the variants EV− and EV+ differ only in the point probability of the fourth-choice option while the choice-outcome contingencies of the remaining three options are identical. For example, for both contexts 3:EV− and 3:EV+, option 1 returns a red point with 0.8 probability, and options 2 and 3 return a red point with 0.1 probability each. The one different option is number 4, where in variant EV+ a red point is received every time, but never in variant EV−. This specific construction of context pairs has the effect that if an agent knows that the current context is context 3 (high posterior probability is associated with that context) but does not know its variant (EV− or EV+), option 1 has the highest expected reward (0.8 red points) of all options while the expected reward for option 4 is only 0.5 red points. This setup makes behaviour of an agent that balances instrumental value with information gain distinguishable from an agent that bases its choices only on the instrumental value (i.e., an agent that maximises expected reward). The agent that infers only the context pair but not the variant, e.g., number 3, will try to maximize expected reward by choosing the option number 1, whereas the agent that also aims to reduce its uncertainty about current context (variant EV− or EV+) would choose option 4. As in real life, sometimes goal-directed exploration pays off, and if an agent that takes into account information gain finds itself in one of the three context variants EV+, it will outperform an agent with purely exploitative behaviour, because it will correctly infer the context and consequently select the option with highest return. However, in the three context variants EV−, an agent maximising instrumental value only will not differentiate between context variants and stick with the second-best option. Consequently, it will collect on average more reward (i.e., has reached more often the goal of collecting four points at the end of a segment) than an agent that also takes into account information gain hence tries to reduce context uncertainty.

Therefore, this nontrivial task design gives an inherent advantage to the agent who can adapt its exploratory tendencies (reliance on epistemic value, that is, information gain) depending on its beliefs about the current context and context variant. Furthermore, if the meta-control enabled agent can predict the moment of change and the upcoming context variant, this will allow it to adjust its behaviour in anticipation of the otherwise hidden change of context.

The following is our attempt to demonstrate that, given the task, we can build a probabilistic inference agent that balances between its exploratory and exploitative tendencies, depending on the beliefs about contexts and meta-control states at the upper level of the hierarchy. Importantly, agents doing the task will learn task parameters during a training period, just as human participants would do. Specifically, agents will have to learn the outcome probabilities (blue point, red point, and no point) associated with each option, in each of the six contexts to be successful in the task and the likelihood of successfully completing a segment in different meta-control states given different contexts. The agent is informed that there are only six different contexts and that the context might change, on average, every five segments, as we fix the model parameter capturing context switch frequency. Initially, we will consider a hidden Markov model for representing context dynamics, similar to related work in sequential inference problems (FitzGerald, Hämmerer, Friston, Li and Dolan, 2017; Schlagenhauf et al., 2014). Note that the hidden Markov model implicitly assumes maximal unpredictability of the moment of change, for a known change frequency. It often is the case in sequential decision making tasks used in cognitive neuroscience that the moments of change are actually unpredictable (Meyniel, Maheu and Dehaene, 2016). However, once one introduces temporal structure to the moments of change, the framework of hidden semi-Markov models is better suited than HMMs to represent this temporal structure and use it for predicting moments of change. Therefore, in a subsequent set of simulations, we illustrate anticipatory meta-control by providing the agent with beliefs about the durations between subsequent changes using semi-hidden Markov models (Marković et al., 2019).

### Behavioural model

We constructed the task such that the context is a hidden variable, which is not directly observable but can be inferred with varying certainty depending on the observed outcomes and the specific sequence of actions the agent performs. As the agent cannot directly observe the underlying hidden states, e.g., which of the six contexts is the current one, the agent has to form beliefs over possible contexts and make decisions based on these beliefs, and thereby resolving the exploration-exploitation dilemma. This means that the decisions of the agent are made under uncertainty about the current context. To define an agent, we will use a two-level deep temporal model as described above.

We depict the hidden states and observables (random variables) as circles in the factor graph shown in Fig. 1. We use ${x}^{\prime \prime }$ to denote hidden states at the second level of the hierarchy and ${x}^{\prime }$ to denote hidden states at the first level. Similarly, ${o}_k$ denotes observations (evidence) at the second level of the hierarchy, which is defined as a binary variable (success or failure), and ${o}_{1:T}=\left({o}_1,\dots, {o}_T\right)$ a sequence of observations at the first level of the hierarchy. At any trial *t* an observation *o*_*t*_ at the first level of the hierarchy consists of three factors:

- point type *f*_*t*_ ∈ {0,1}^2^,
- total number of points of each type *w*_*t*_∈ {0,…,5}^2^,
- selected option *l*_*t*_ ∈ {1,…,4}.

Hence *o*_*t*_ = (*f*_*t*_, *w*_*t*_, *l*_*t*_). Note that the point type *f*_*t*_ is expressed as a two-dimensional vector (Null – ( 0, 0), Blue – (1, 0), Red - (0, 1)) and the total number of points *w*_*t*_ is obtained as

$$ {w}_t={f}_t+{w}_{t-1}={w}_0+\sum \limits_{n=1}^t{f}_n $$

where *w*_0_ = (0, 0). At the lower level of the hierarchy the hidden states ${s}_{1:T}^{\prime }$ consist of the following factors (${l}_t^{\prime },{i}_k^{\prime },{c}_k^{\prime }$), selected option, auxiliary meta-control state and auxiliary context. Note that ${i}_k^{\prime },{c}_k^{\prime }$ are constant variables at the lower level, which are linked to the dynamic counterparts on the upper level. The auxiliary variables are necessary to guide the learning of the observation likelihood *A*^′^, and policy selection at the lower level. At the upper level of the hierarchy, hidden states ${s}_k^{\prime \prime }$ factorise into context ${c}_k^{\prime \prime }$, context duration ${d}_k^{\prime \prime }$, and meta-control state ${i}_k^{\prime \prime }$, hence ${s}_k^{\prime \prime }=\left({c}_k^{\prime \prime },{d}_k^{\prime \prime },{i}_k^{\prime \prime}\right)$.

The agent’s generative model of the task represents the known probabilistic mappings between hidden states, their transitions, and outcomes. We will assume fixed state transition probabilities, $p\left({s}_k^{\prime \prime }|{s}_{k-1}^{\prime \prime },{\pi}^{\prime \prime}\right)$ and $p\left({s}_k^{\prime }|{s}_{k-1}^{\prime },{\pi}^{\prime}\right)$. In other words, the agent has a predefined knowledge about the state transitions at both levels of the hierarchy. In contrast, the beliefs about state outcome probabilities, $p\left({o}_k|{s}_k^{\prime \prime },{A}^{\prime \prime}\right)$ and $p\left({o}_t|{s}_t^{\prime },{A}^{\prime}\right)$, are not known a priori and are learned throughout the task by updating beliefs about state-outcome contingencies *A*^′^ and *A*^′′^, which define the likelihoods. Hence, the agent will learn to associate each of the six contexts with a specific probabilistic option-outcome mapping (Fig. 2).

Importantly, to model beliefs about context dynamics, we will use hidden semi-Markov models (Yu, 2010) that combine beliefs about hidden states (${c}_k^{\prime \prime }$ ) with beliefs about their duration (${d}_k^{\prime \prime }$). In practice, this modelling choice allows us to explore how the precision of beliefs about the hidden moment of change interacts with meta-control capabilities of the agent. Here, we will use the representation of the hidden semi-Markov model also for simulating behaviour under the hidden Markov assumption, as this represents a special case of the semi-Markovian state change representation (see *Methods* for more details).

To reach the segment-wise goal of collecting at least four points of a single colour, the agent has to plan ahead and select behavioural policies, i.e., sequences of actions. When the agent has learned the relation between states and outcomes at both levels of the hierarchy, the agent can make predictions about the consequences of selecting a specific policy, within each segment at the lower level of the hierarchy and between segments at the upper level of the hierarchy. Importantly, the policy selection at both levels is defined as minimisation of the expected free energy (EFE). Minimising EFE corresponds to maximising the expected instrumental value (IV), i.e., the expected amount of reward and maximising the epistemic value (EV), that is, the information gain (see e.g., Kaplan and Friston, 2018). Note, that at the lower level of the hierarchy the instrumental value is proportional to the expected probability of collecting 4 points of a single colour at the end of the segment and at the upper level to the expected probability of succeeding in the next segment. As outlined in the previous section, we have designed our task such that the fourth option in each context (Fig. 2) carries the highest information gain, because it clearly differentiates between the context variants EV− and EV+. In contrast, the expected instrumental value of fourth option is relatively low compared with the second-best options, when the context variant is not known.

### Simulations

We will proceed with simulations in three stages. First, to illustrate the basic features of the model, we will show the behaviour of agents that are fixed in their balance between information gain and expected reward, that is, between an explorative versus exploitative stance. Hence, these agents do not perform meta-control. Second, we will introduce adaptive agents that can supress or boost its exploratory tendencies, hence resolve the exploration-exploitation dilemma by adapting its meta-control states in a context-dependent fashion. Third, we will show that by utilising the framework of hidden semi-Markov models we can introduce anticipatory behaviour enabling an agent to change its meta-control state in anticipation of a predicted context switch.

**Fig. 3:**
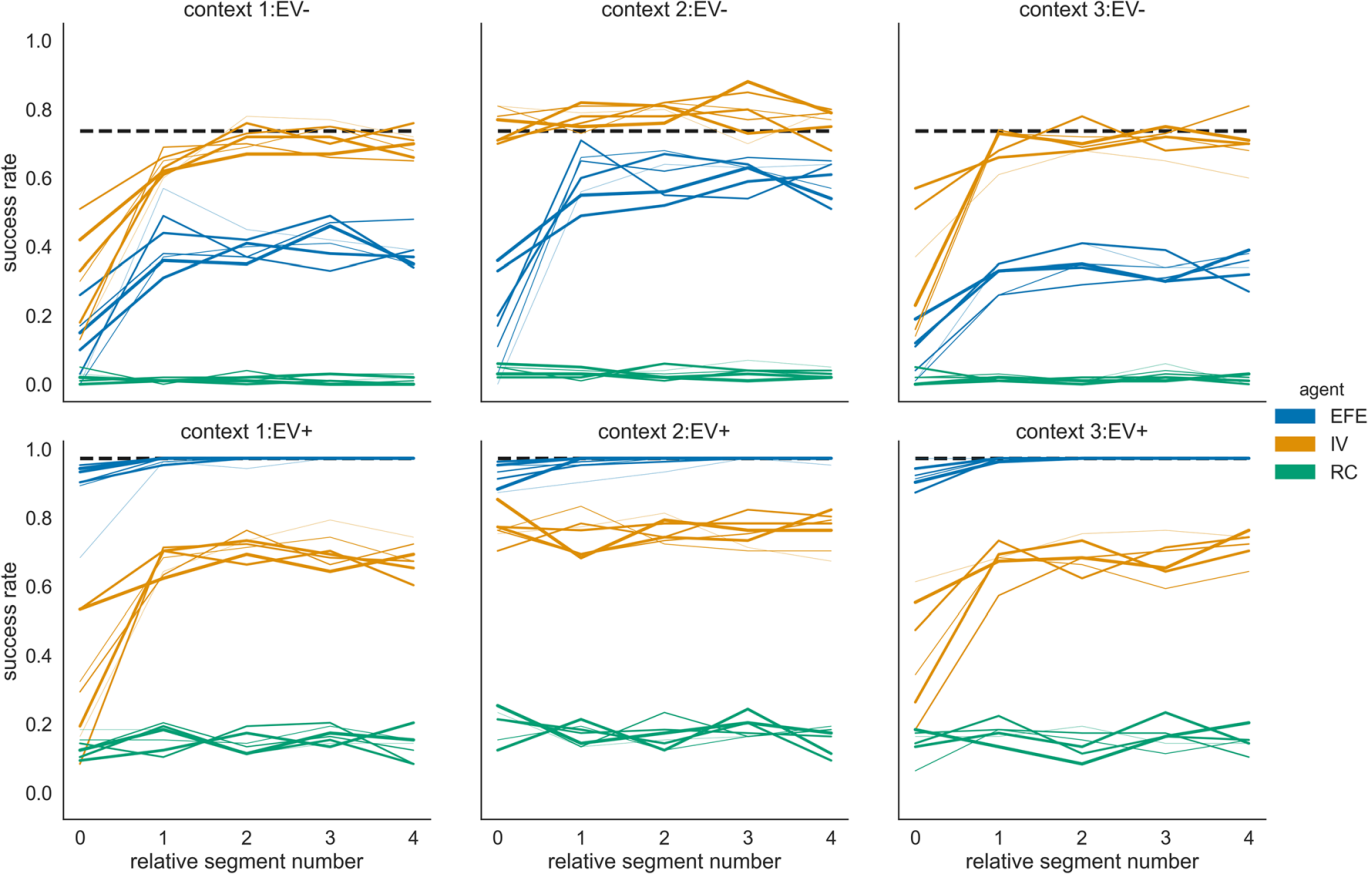
Success rates of three different agents in six different contexts. Group mean success rates for the expected free-energy agent (EFE; blue lines), instrumental agent (IV; orange lines), and a random choice agent (RC; green lines), which randomly selects one of the four options on a trial with equal probabilities. The black dashed line denotes the expected success rate for always selecting the option that returns a coloured point with the highest probability. We use thinner lines to mark mean success rates in early context blocks and thicker lines for context blocks later in the experiment.

In the first illustrative simulation, we exposed agents to the task for 200 segments, i.e., 1,000 trials. In Fig. 3, we show group mean success rates of three different agent types, where each group consists of *n* = 100 agents of the same type. One of these agents simply serves as a reference random choice (RC) agent. The other two agent types differ in their policy selection objective. In one case, the policy selection objective corresponds to the instrumental value (IV) only and in the other case to the expected free energy (EFE), i.e., the combined instrumental and epistemic value. In the task, maximizing IV only results in exploitative behaviour of an IV agent while an EFE agent is expected to show goal-directed exploration because of the EFE’s epistemic value component. We assume that the two agents have sufficiently learned the choice-outcome probabilities for the six contexts after 100 segments. Note that we used an alternating pattern of context variants EV− and EV+ to maximize the need for adapting to a new context, see also below. As expected due to the task design, there are large performance differences between context variants EV− and EV+. This is because in context variants EV+, for each of the three contexts, there is the fourth option that returns a point with certainty, see the task description above. The EFE agent reaches the highest performances in variants EV+, because the affinity toward informative choices enables the agent not only to resolve the uncertainty about the current context but also to collect points with maximal probability. In EV− variants, the EFE agent has clearly a worse performance than the IV agent.

**Fig. 4:**
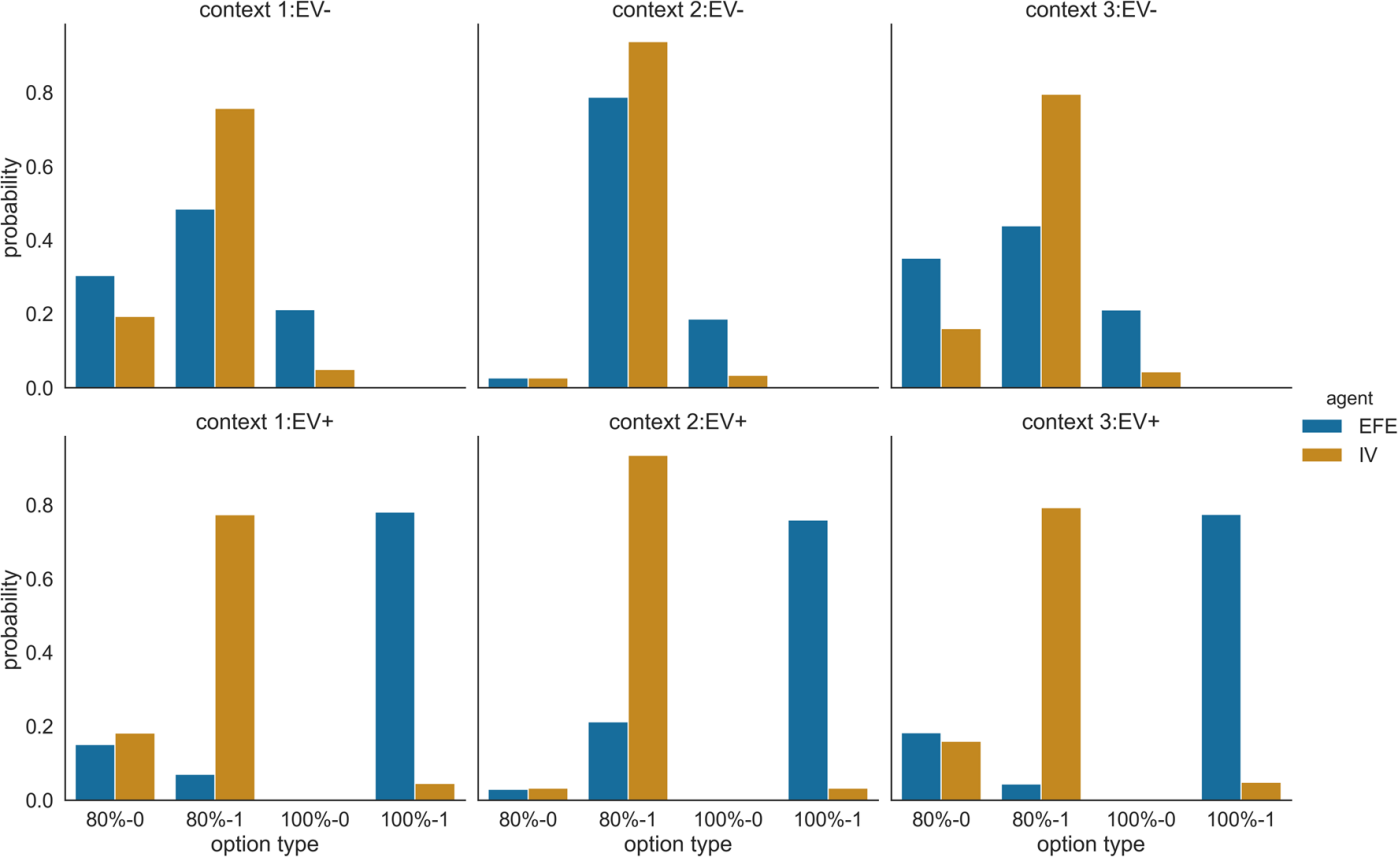
Probability of selecting different options in different contexts and context variants. The probabilities are estimated from 100 simulations for the IV and EFE agents and pooled across the last 100 segments of the experiment. The three context variants EV*−* are shown in the upper row and the three context variants EV+ in the lower row. The EFE agent (blue bars) selects the informative options (option type 100%-1) with the highest rate when exposed to variant EV+, and is also more likely to select the informative option (option type 100%-0) when exposed to variant EV*−* compared with the IV agent (orange bars). For visualisation, we have pooled options that return a point with high probability (independent of the colour, 80%-1 and 100%-1) and options that return no points with high probability (80%-0 and 100%-0).

To understand the difference between the mean success rates of the IV and EFE agents in both context variants EV− and EV+, we now take a closer look at their choice probabilities. In Fig. 4, one can see that the EFE agent is more likely to select the fourth option, which is the most informative about the current context (Fig. 2a). This allows the agent to resolve uncertainty about the context rapidly, leading to higher performance in EV+ context variants and lower performance in EV− context variants as initial trials are used to reduce context uncertainty and identify the true context.

### Adaptive control of the exploration-exploitation dilemma

Note that the relative contributions of the instrumental and epistemic value to the policy selection were fixed in both the IV and EFE agent. However, one could argue that agents should be able to adapt their behavioural mode depending on the context, i.e., use autonomously controlled contributions of the two value terms for policy selection, akin to human meta-control.

We implemented the conceptual idea to enable such meta-control in an agent by linking the inference over meta-control states, which define contributions of the instrumental and epistemic values to policy selection. These meta-control states ${\mathrm{i}}_{\mathrm{k}}^{\prime \prime }$ are part of the second level states ${\mathrm{s}}_{\mathrm{k}}^{\prime \prime }$ (see the graphical model in Fig. 1) and linked to each context via observations of success or failure in each segment. Specifically, the meta-control states adapt the selection of policies by changing the prior over policies at the lower level that is proportional to the expected free energy, see also (Parr and Friston, 2019). Intuitively, the prior over policies can be interpreted as a behavioural mode or a strategy, because the prior simply tells an agent which action sequences it should currently prefer. Importantly, the prior over policies, depending on the meta-control state, will either take the epistemic value term into account or ignore it. However, the uncertainty over the currently preferred meta-control states will lead to a continuous weighting of the epistemic value term. The adaptive weighting biases the set of a priori viable policies, by downscaling or upscaling the information gain (see Eq. (1) and Priors over policies – expected free energy for more details), which in turn influences the computations of the posterior over policies. We anticipate that such an adaptive agent will learn to be biased towards exploitative behaviour in context variants EV− and towards explorative behaviour in context variants EV+. In other words, an observer of the agent’s behaviour would possibly conclude that this agent resolves the exploration-exploitation dilemma by exerting meta-control.

Critically, the meta-control states do not represent external states of the environment but rather internal modes of behaviour. Note that the prior over policies does not exclude any policies in a hard-wired fashion. Rather, some policies become more likely to be selected than others.

**Fig. 5:**
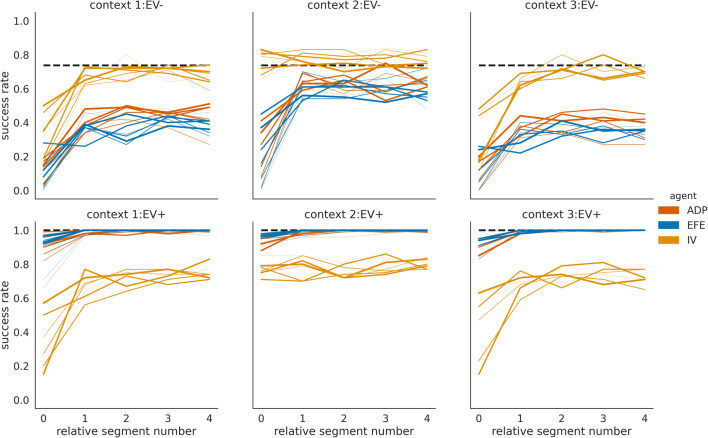
Success rates of an adaptive (controlled) and two non-adaptive agent types. Group mean success rate for 100 agents of the adaptive (ADP), EFE minimising (EFE), and IV maximising (IV) agent type, plotted over the second half of the experiment. The horizontal black dashed line denotes the expected mean success rate for always selecting an option which returns a coloured point with probability *p* = 0.8. Note that the success rates of the adaptive and the EFE agents are similar in the context variants EV+ as the mean performance overlap. The black dashed line denotes the expected success rate for always selecting the option which returns a coloured point with highest probability. We use thinner lines to for early context blocks and thicker lines later five blocks of the same context.

To show this, we will compare the behaviour of this adaptive agent (ADP) to the behaviour of the IV and EFE agents, which we used in the simulations above. These two nonadaptive agents represent the two extreme modes of the adaptive agent: the IV agent corresponds to a zero weighting of the information gain, and the EFE agent to the unit weighting of the information gain (see Priors over policies – expected free energy for details). In Fig. 5, we show the group mean success rates of the adaptive and the two nonadaptive agents, using the same task design (as shown in Fig. 2). One can see that the adaptive agent is on average similar in performance to the EFE agent in the context variants EV+, which shows that the adaptive agent increases the weight of the information gain in EV+ context variants. However, in context variants EV−, the performance of the adaptive agent is slightly better compared with the EFE agent (but still far below the IV agent).

**Fig. 6:**
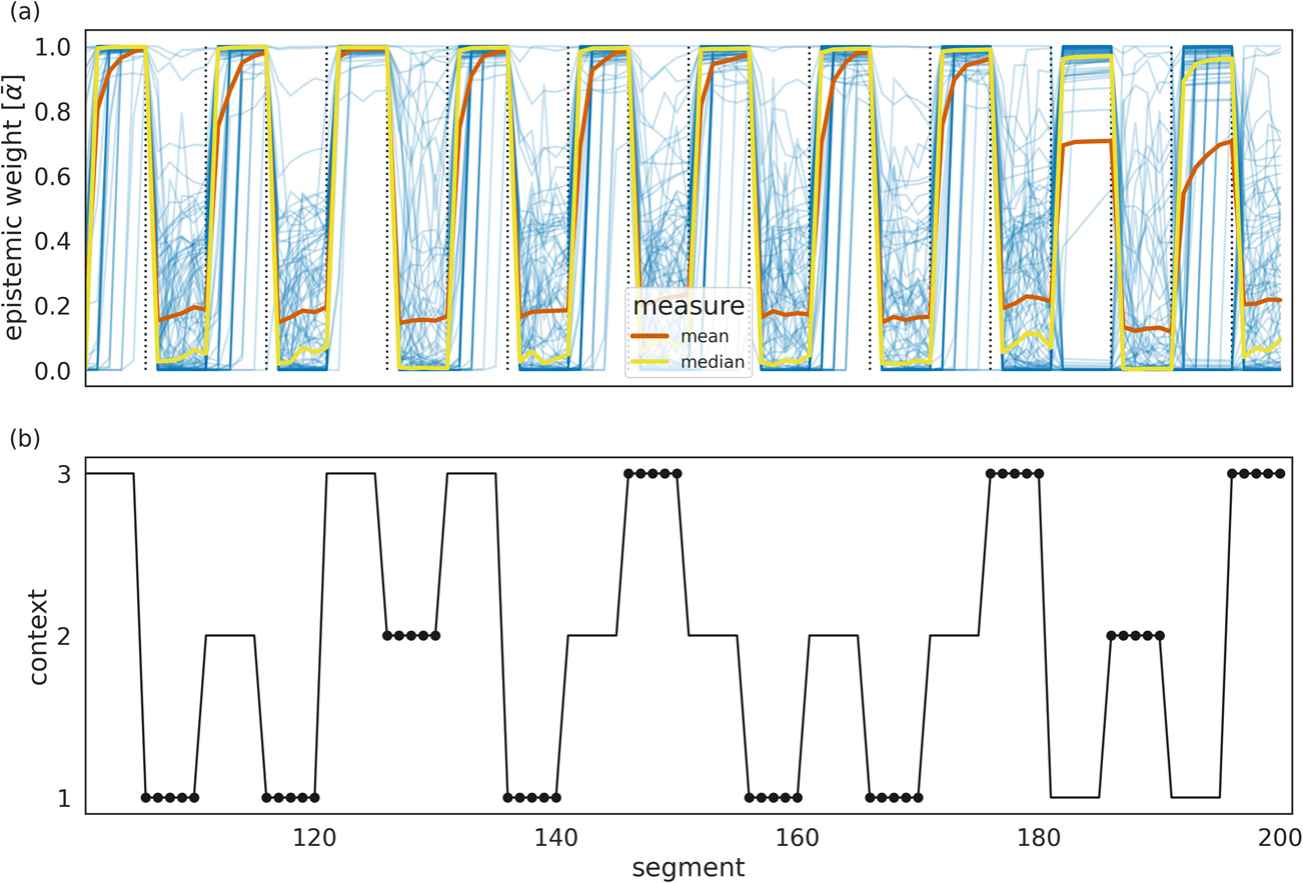
Adaptive weighting of the information gain. (**a**) Trajectories of the weighting $\overline{\alpha}$ of the epistemic value contribution to the policy selection. The closer this value is to zero the more exploitative the agent becomes. To show the variability of the 100 agents’ individual $\overline{\alpha}$ trajectories, we plotted the median $\overline{\alpha}$ trajectory (yellow), the average $\overline{\alpha}$ trajectory (red), and the individual $\overline{\alpha}$ trajectories (blue). (**b**) For comparison, the context change dynamics limited to the last 100 segments of the simulated experiment.

The reason for this apparent nonadaptation to the context variants EV− are shown in Fig. 6a, where we plotted the trajectories of the weighting $\overline{\alpha}$ of the epistemic value for policy evaluation, i.e., a value of 1 indicates that the adaptive agent balances information gain with instrumental value, whereas a value of 0 indicates policy selection based only on the instrumental value. Due to the learning in the first half of the experiment, the dynamics of the weighting factor $\overline{\alpha}$ are history dependent, as can be seen for the trajectories of 100 agent instances doing exactly the same task with the same context sequence but with differently sampled outcomes (see Fig. 6a, blue lines). This implies that the stochasticity of the outcomes interacts with the learning process on both levels of the hierarchy generating unique, adaptive behaviour that is sensitive to previous experience.

**Fig. 7:**
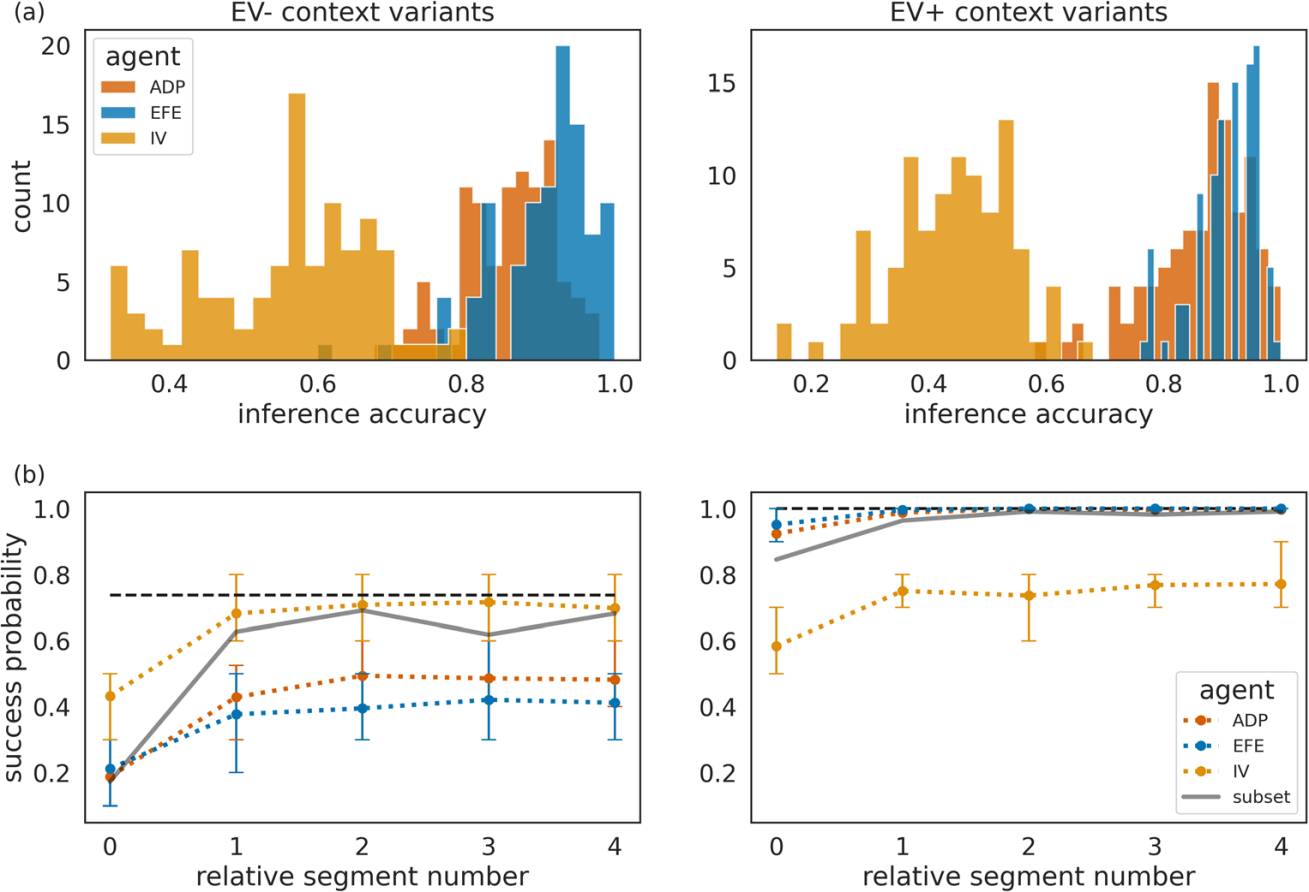
Quantification of between-agent differences in group context inference accuracy and group mean success rates. (**a**) Context inference accuracy histogram for the two contexts variants A and B, for the adaptive (green), exploratory (orange) and exploitative (violet) agent type, estimated over the last 100 segments of the experiment and defined as group probability of assigning the posterior mode to the current context. (**b**) Average success probability estimated over *n* = 100 instances of each agent type, over the last 100 segments of the experiment. We used the last 100 segments of the experiment to estimate success probability per instance of each agent type. The relative segment number denotes the segment number relative to the moment of context change, where zero corresponds to the segment at which the context changed. The error bars show the 25^th^ and the 75^th^ percentile. The same colour scheme as in (a) applies, where in addition, we show as black solid lines the average success probability of a subset of 10 instances of the adaptive agent which were the most efficient in down-regulating exploratory behaviour (see text for more details).

To further quantify the differences between the adaptive agent and the two nonadaptive agents, we looked at two other quantities: (i) The context inference accuracy (Fig. 7a), defined as the probability that the agent correctly identifies the current context (measured by the highest posterior probability for the true context). The adaptive agent achieves high levels of inference accuracy in both context variants. In other words, the adaptation of the behavioural modes does not have a detrimental impact on the ability of the adaptive agent to resolve its uncertainty about the current state of the world. This is in contrast to the IV agent, which on average has inaccurate beliefs about the current context. Note that the IV agent also is capable of exploration, albeit only random exploration, which on average leads to a lower information gain. (ii) The success probability of different agents computed over all repetitions of the same context (Fig. 7b). In context variants EV+, the success probability of the adaptive agent is as high as the success probability of the EFE agent. However, in context variants EV−, the adaptive agent’s success probability is lower compared with the one of the IV agent, but significantly higher than the EFE agent (*p* < 0.05 as per Wilcoxon signed-rank test for all relative segment values). This lower performance of the adaptive agent compared with the IV agent can be directly related to the wide distribution of trajectories of the weighting factor $\overline{\alpha}$ as shown in Fig. 6a. Many of the 100 adaptive agents, due to the high stochasticity of the task (probabilistic sampling of outcomes), do not learn how to behave exploitatively in context variants EV−. This point of variability in experience-dependent adaptation is stressed by showing the average success probability of a subset of ten instances of the adaptive agent, which learned to down-regulate $\overline{\alpha}$. We selected these ten agent instances using the criterion of a downregulated epistemic weight below the 0.5 level in context variants EV−. One can clearly see (Fig. 7b, grey line) that the average success probability of this subset of adaptive agents is close to the performance level of the exploitative agent.

Therefore, the overall low group mean performance of the adaptive agent in the EV− context variants may be explained by the difficulty of downregulating exploratory tendencies in the presence of various sources of uncertainty. This is because the adaptive agent has to update continuously its beliefs about the current context, choice probabilities, and relations between the meta-control states, contexts, and the success probability for a segment. In other words, the adaptive agent works as expected, but the stochasticity of its task environment keeps the adaptive agent in a limbo of uncertainty and drives the agent often into an exploratory mode. This suggests that the adaptive agent could fare better in our task environment if we reduced the agent’s overall uncertainty by providing it with a more accurate representation of changes in the task environment.

So far, we have limited the agent to an imprecise prior on when to expect a context change, i.e., an agent expects a change after each segment with probability *p* = 1/5. It is reasonable to assume that a human participant would learn after an extended period of 100 segments (500 trials) that there might be a context change around every 5 segments, where the stochasticity of the task still makes the exact duration of a context difficult to predict, but at least there should not be an anticipation that there is a context change after each segment. If we gave such a prior about the duration between context switches to an adaptive agent, it would anticipate the moment of change and maintain high precision on the current context for a longer time. In the next section, we will show how representing the moment of change can improve the performance of adaptive agent and bring it much closer to the performance of the exploitative agent (IV agent) in EV− context variants.

### Anticipatory control of behaviour

The agents described so far were limited to expecting context change in every segment with a constant switch probability (of *p* = 1/5), corresponding to a standard hidden Markov model. Here, we enable agents to represent the temporal structure of the task better and anticipate a switch around every five segments: to understand how introducing temporal representations drives anticipatory behaviour we will not consider a precise prediction of a switch after five segments, but a low uncertainty over possible durations between subsequent changes, see *Methods* for details. We introduce temporal expectation about changes by providing the agent with a more precise prior over context durations, that is, the number of segments before the next change occurs. This representation corresponds to replacing the hidden Markov framework with the hidden semi-Markov framework (Marković et al., 2019).

**Fig. 8:**
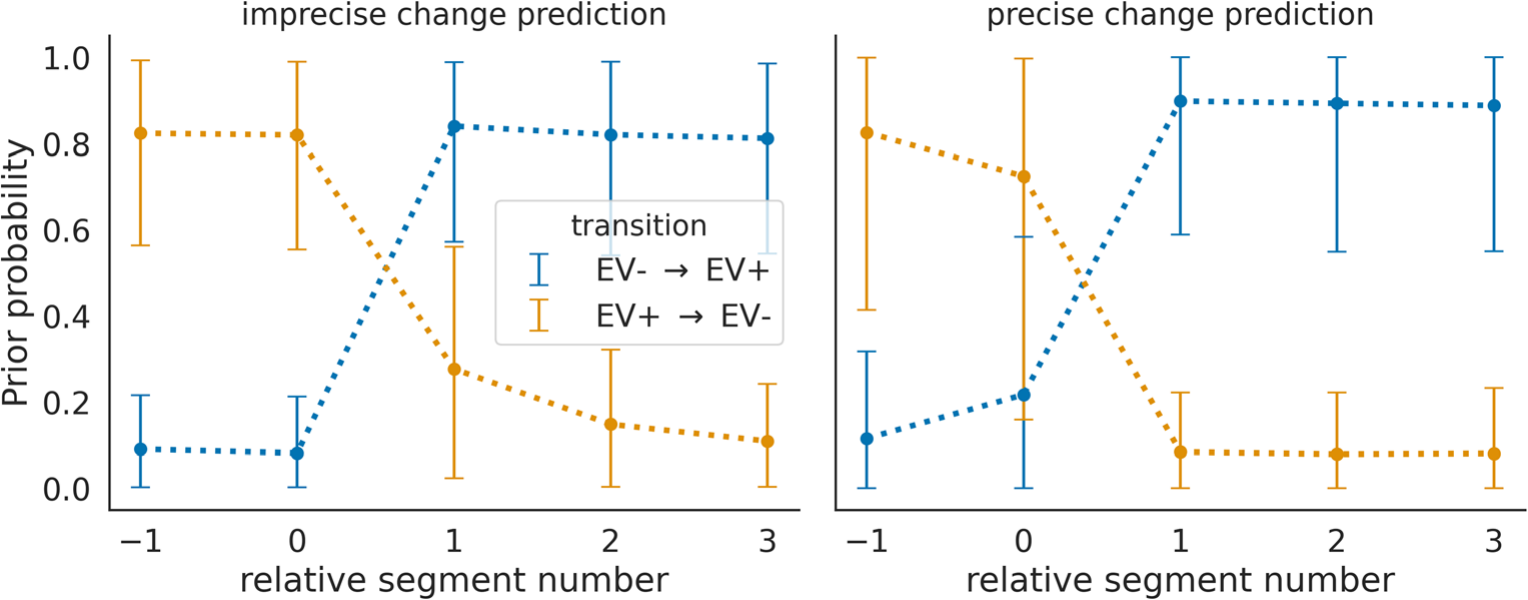
Modulation of prior beliefs over meta-control states by the anticipation of upcoming context change. (**Left**) The adaptive agent with imprecise change prediction, where prior probabilities over meta-control states during two types of transitions are plotted. These prior probabilities are entertained by the agent after the end of a segment before observing the outcome of the first trial of the next segment. One transition type changes from a context variant EV− to EV+ (blue), the other from a context variant EV+ to EV− (orange). The solid lines denote the mean, estimated over multiple transitions between two context variants, and the error bars show the 10^th^ and 90^th^ percentile. (**Right**) The agent with precise prediction, in comparison to the agent with imprecise prediction, adapts its prior belief over the meta-control state before having seen evidence for this change. This can be seen by comparing the prior probabilities of the two agents at relative segment number 0. One can also see that the agent with precise prediction has on average more extreme prior probabilities (closer to 0 and 1). This indicates that precise change predictions also enables the adaptive agent to gain more certainty about the current behavioural mode.

If the adaptive agent can form predictions about the moment of change, it can use that prediction to adapt its meta-control states and any control signal a priori, before observing outcomes of the upcoming segment. To illustrate this, we show in Fig. 8 prior beliefs about the meta-control state (which is represented by the weighting factor $\overline{\alpha}\Big)$ for two variants of an adaptive agent, one with imprecise predictions as we used in the simulations above, and one with precise predictions, see *Methods* for details. Importantly, one can see that the agent with precise predictions also changes its prior beliefs about its meta-control states when anticipating change (i.e., at the relative segment number 0 the group mean prior beliefs are reduced already before the change was observed in terms of outcomes). In contrast, the agent with imprecise predictions (i.e., the adaptive agent described above with a constant switch probability of *p* = 1/5) changes its prior beliefs only after interacting with the environment and observing a change of context at relative segment number 1.

**Fig. 9:**
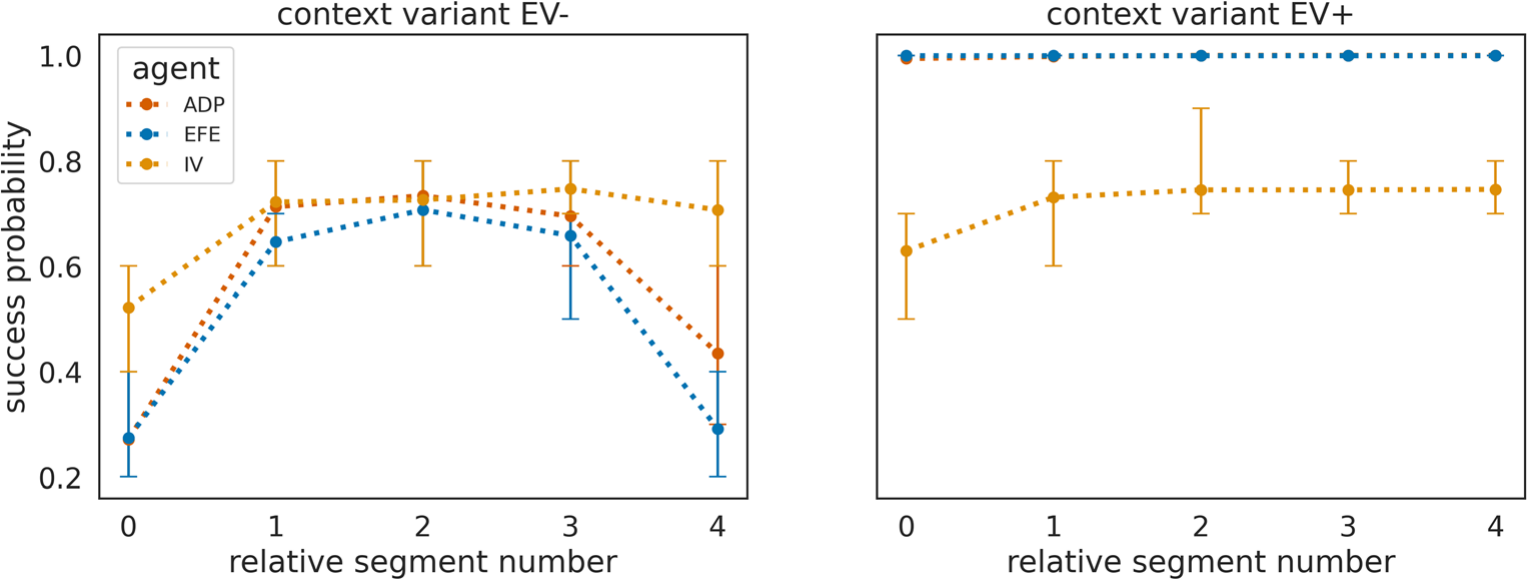
Success probability of three different agent types with strong change prediction. Mean success probability estimated as the average of success probabilities of *n* = 100 instances of each agent type in (**left**) context variants EV*−* and (**right**) in context variants EV+. Note that in context variant B the adaptive agent (green line) shows the same mean success probability as the explorative agent (orange line) so that the green line is hidden from view. We used the last 100 segments of the experiment to estimate success probability relative to the moment of change. The relative segment number denotes the segment number relative to the moment of context change, where zero corresponds to the segment at which the context changed. The error bars show the 25^th^ and the 75^th^ percentile.

How does the precise prediction of context changes modulate an agent’s performance in the two context variants EV− and EV+? In Fig. 9, we show a comparison of success probabilities of the three agent types. As expected, we find that all agent types benefit from precise predictions of context changes, in comparison to imprecise predictions, as shown in Fig. 7b. In context variant EV−, we find a significantly higher performance (*p* < 0.05 per Wilcoxon signed-rank test) of the adaptive agent, relative to the EFE agent, for relative segments 2, 3, and 4. We expect that increasing the number of instances (simulations) will trivially lead to significant differences for all comparisons. Furthermore, as the higher average performance of the adaptive agent is stable over repeated simulations (data not shown), we can exclude a chance occurrence of performance differences. In contrast to adaptive agents with imprecise change prediction, we find that with a precise change prediction the majority of agent instances (90 of 100) down-regulate the use of epistemic value in context variants EV− (<0.5 level as above). Note that the IV agent is insensitive to the epistemic value (information gain) and therefore does not base policy selection on its subjective uncertainty about the current context. As a consequence, the IV agent will stick with the less informative options and have a higher chance of succeeding in context variants EV−. This becomes obvious for the relative segment 0 in Fig. 9, where the adaptive and EFE agents aim at reducing context uncertainty and at relative segment 4 just before another context change. Here, although the two agents have a strong prior for change prediction, they still expect the change with some probability at relative segment 4 already so that they experience increased uncertainty about their current context.

**Fig. 10:**
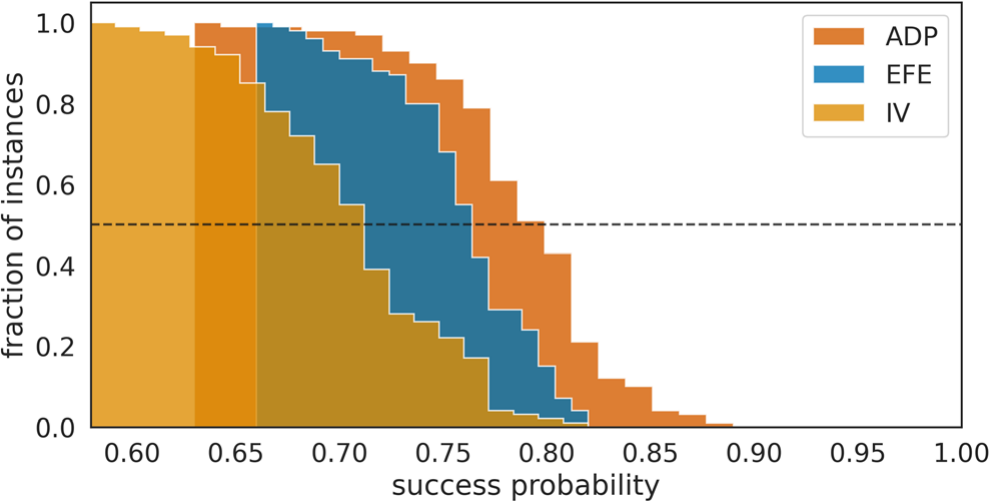
Survival function of success probabilities. Survival function (i.e., complementary cumulative distribution) for three different agent types with precise change prediction, using the same simulations as in Fig. 9 over the last 100 segments of the experiment. We pooled across the two context variants EV*−* and EV+. The adaptive agent (ADP) has the highest chance of generating a high performing instance over most success probabilities.

Another view at the results shown in Fig. 9 is to not focus on the differences in mean success probabilities, as one would in the analysis of a psychological experiment, but to evaluate agent performance from a competitive “survival of the fittest” perspective. The question is then what agent type, after an initial learning period, has the highest chance to produce the best-performing agent instances: the nonadaptive or the adaptive, controlled agent? In Fig. 10, we show the survival function of cumulative successes of the three agent types with precise change predictions (ADP, EFE, and IV). The survival function is estimated over *n* = 100 simulations of each agent type, and as in Fig. 9, we used the last 100 segments (where we pooled over context variants EV− and EV+) of the experiment to estimate success probability per instance. Critically, we found that 50% instances of the adaptive agents achieved a success probability ≥80%, leading to the largest probability of observing a high performing adaptive agent instance among the three agent types. For example, in an environment where an agent requires at least an 80% success probability to survive, this world would be populated mostly (66%) by adaptive agents (i.e., agents with meta-control).

## Discussion

We have proposed a model that casts meta-control as an arbitrating, context-specific mechanism underlying planning and decision making under uncertainty. We used the example of the exploration-exploitation dilemma to illustrate how an agent adapts its behavioural modes (encoded as the priors over policies), i.e., its internal preferences to specific sequences of actions. Critically, the agent arbitrates between explorative and exploitative behaviour by changing the relative weight of epistemic value (expected information gain) relative to the instrumental value (expected reward) when evaluating the posterior probability of different policies. As we have shown, this context-specific weighting results in adaptive transitions between explorative or exploitative behaviour, depending on the context inferred by the agent. The key element of the proposed model are meta-control states, which encode the different modes of behaviour, and can be used to learn the association between contexts and appropriate modes of behaviour. We have shown that inference over meta-control states and control signals (which make the agent behave according to its specific meta-control states) leads to adaptive meta-control as a function of the agent’s beliefs about the current context.

Various experiments utilizing restless multi armed bandits typically vary the mean payoffs over trials, e.g., (Daw and Doya, 2006; Speekenbrink and Konstantinidis, 2015) where the considered behavioural models implement different strategies of how humans may balance exploitative and exploratory actions. Experiments based on these dynamic environments ask the question how humans sample the different arms to stay on top of relative changes of mean reward rates (Speekenbrink and Konstantinidis, 2015). However, in an everyday environment, we often experience situations (contexts) that incentivise or punishes either exploration or exploitation. Furthermore, we have often already learned what behavioural modes are the best for specific contexts. Hence, a typical problem is not so much to compute the balance between exploration and exploitation but rather to identify the current context and apply the previously learned balance for that specific context. In such a setup, the problem of deciding which behavioural mode to use is reduced to identifying the specific context we just got into. Hence, we expect that the better agents are in reducing the uncertainty about various aspects of the environment (hidden states and their dynamics), the better they will be in controlling their behaviour. In the present toy example, we show that the context-dependent level of exploration can be learned and inferred in an online fashion. Note that the model currently does not make much use yet of the hierarchical architecture, e.g., by introducing context change dependencies. Such an extension would allow agents to plan and navigate within the contextual space itself and can be applied to future experiments. Although we did not present a behavioural experiment, we would expect that the precision of participants’ beliefs about the current context and the anticipation of context changes will be inversely proportional to their reaction times. More generally, the hierarchical representation of control-dilemmas can help us to relate representational (belief) uncertainty at different levels of the hierarchy to specific features of human decision making, such as reaction times, choice certainty, and specific behavioural strategies.

### Related approaches and algorithms

Two components of any decision-making algorithm are central to the resolution of the exploration-exploitation dilemma: (i) an inference (learning) algorithm which defines how an agent updates their beliefs and learns to represent the latent dynamics based on a sequence of performed actions and observed outcomes; (ii) an action selection algorithm which defines how an agent chooses next action based on its current beliefs. How humans learn a latent dynamical structure and update their beliefs about a changing world under various sources of uncertainty (Dayan and Angela, 2003) has been a key topic of neuroscience research for at least a decade (Behrens, Woolrich, Walton and Rushworth, 2007; Doya, 2002; Mathys, Daunizeau, Friston and Stephan, 2011; McGuire, Nassar, Gold and Kable, 2014; Meyniel, Sigman and Mainen, 2015; Nassar, Wilson, Heasly and Gold, 2010). Although we illustrate learning and inference from the perspective of variational inference, in practice, any learning algorithm and an approximate inference scheme would be viable and may be related to active inference and minimisation of the expected free energy. The critical point to establish this relationship is to define action selection as a balance between maximisation of expected reward and expected information gain, which are functionals of the posterior beliefs about latent states of the world.

Therefore, given an approximate inference scheme (which comprises the learning algorithm) one can define action selection either as direct minimisation of the expected free energy, or as an approximation of that process. For example, commonly used algorithms, such as upper confidence bound (UCB) (Garivier and Cappé, 2011) and Thompson sampling (Agrawal & Goyal, 2012), can be seen as specific approximations to the process of minimising expected free energy, which balances expected value with both random and directed exploration (Schwartenbeck et al., 2019). The combination of random and directed exploration is an important feature of human behaviour (Wilson et al., 2014). The UCB algorithms rests on balancing the expected reward and the confidence bound, which can be seen as an approximation to the expected information gain. Similarly, Thompson sampling or random exploration can be obtained in the limit of low action precision (small *γ*). Although a direct comparison between UCB, Thompson sampling, and active inference would clarify their correspondence, we leave such comparison for future works focused specifically on establishing the relation between different approaches.

Finally, we would like to stress that the take-home message from our results for the emergence of meta-control between exploration and exploitation is not how exactly the update equations are implemented but rather that a hierarchical representation is employed. Furthermore, this type of meta-control rests upon the interaction between different levels of the hierarchy, the reduction of uncertainty about latent states of the world, meta-control states, and actions (policies) at different levels of the hierarchy, and finally selection of hierarchy-specific control signals which balance expected value and expected information gain.

### Meta-control: mapping of contexts to strategies

The proposed model describes a way to compute meta-control as a way of associating specific contexts with specific behavioural policies (modes of behaviour). Crucially, this is precisely the way that Heilbronner and Hayden (2016) describe in a recent review the hypothesized function of dorsal anterior cingulate cortex (dACC). In their section “Mapping contexts to strategies,” they write, “We propose, therefore, that the dACC embodies a type of storage buffer that tracks task-relevant information to guide appropriate action.…” We speculate that the inference, that is, evidence accumulation about the meta-control states is implemented in dACC. This would explain why dACC tracks task-relevant information as would be required when inferring the context and the appropriate meta-control state, which is used for guiding concrete behaviour. This view is congruent with proposals that dACC is involved when switching away from the current task set (Collins and Koechlin, 2012; Duverne and Koechlin, 2017; Gruber, Diekhof, Kirchenbauer and Goschke, 2010) or an ongoing task (Kolling, Behrens, Mars and Rushworth, 2012), where the idea is that dACC does not only represent the ongoing context, including task-relevant states and prior over policies but also potentially relevant alternative contexts and in particular their associated prior over policies.

In the proposed model, the representation of the current and potentially relevant alternative contexts is the only way the agent can infer, when faced with uncertainty about the current context, the appropriate setting of the meta-control states. In other words, the reason why dACC seems so involved in representing task-relevant and potentially task-relevant states may be that inference about the current context is typically not straightforward as there are several sources of uncertainty that will obscure context identity and must be routinely resolved by the brain, even in well-controlled experimental settings. It also is important to note that Heilbronner and Hayden refer to “strategies” and describe dACC’s function as “guiding action.” This is important because in the proposed model, meta-control states do not select actions directly but instead modulate the action selection process by adapting the prior over policies. This means that the prior over policies shapes viable behavioural strategies as the prior constrains the space of available policies and supresses selection of policies that were associated with lower performance contexts.

### Control signals

Assuming that dACC guides the action selection process (Heilbronner and Hayden, 2016), it is an open question what control signals are effectively sent to lower motor hierarchies, such as primary motor cortex? For example, Shenhav et al. (2013) argue that the brain should compute a control signal of a specific identity (what is controlled?) and a specific intensity (how strongly?) where it is an open question how these control signals are computed and how they modulate concrete action selection in a given task. It is precisely this sort of quantitative questions that one may address using the proposed model. For example, in Fig. 6a, we show how much the epistemic value contributes to action selection in a specific context and specific trial. These variations directly modulate the prior over policies and can be readily interpreted as a control signal of specific identity (what policies are preferred) and intensity (how high is the prior for each policy). In other words, the proposed model and variants may be used in the future for making testable predictions how strong specific actions are preferred in a given trial, for a specific experimental sequential decision-making task where participants have to plan under uncertainty, in order to reach goals.

### Meta-reasoning as context inference

For artificial agents, another prominent control dilemma has been subsumed under the topic of rational meta-reasoning, i.e., how agents can select a strategy that selects actions in time and strikes a balance between expected computational costs and expected performance (Boureau et al., 2015; Gershman, Horvitz and Tenenbaum, 2015; Lewis, Howes and Singh, 2014; Lieder and Griffiths, 2017). Here, an interesting research question is whether one can reduce this type of meta-control to, as proposed here, context learning and probabilistic context inference. The idea is that previously encountered contexts enable the agent to learn a prior over policies for this context; see Maisto, Friston and Pezzulo (2019) for a recent example for modelling the arbitration between habits and goal-directed control. As we have shown, the agent can learn for each of these contexts a prior over policies, which can be considered the set of default behaviour of an agent in this specific context. If the brain used such a discrete contextual tiling of its environment, phenomena, such as maladaptive habits, where meta-reasoning seems short-circuited, could be at least partially explained by suboptimal context inference, as may be the case in Pavlovian to Instrumental Transfer experiments (Garbusow et al., 2014).

### Beyond exploration-exploitation: extension to other cognitive control dilemmas

The general question of meta-control, i.e.. how humans infer how to make their decisions, results in a wide range of experimentally established cognitive control dilemmas. Three examples of these are (i) the goal shielding-shifting dilemma which relates to a problem a decision maker faces when pursuing a long-term goal in multi-goal settings. To reach a long-term goal, the agent has to ignore (shield) competing goals to prevent premature goal shifts (Goschke and Dreisbach, 2008). However, the agent has still to be aware of the existence of alternative goals as in dynamic environment agent should be able to flexibly switch between goals and adapt behaviour to changing task demands or reward contingencies. (ii) The selection-monitoring dilemma relates to the problem a decision maker faces when deciding to pay attention to a specific part of the environment while trying to reach a goal (Goschke and Dreisbach, 2008). Typically, not all available information is relevant for the task at hand, and paying attention to all of it would be detrimental for performance. However, completely ignoring currently irrelevant information would prevent the agent from noticing a crucial change in the environment and adapting its behaviour. (iii) The anticipation-discounting dilemma relates to the problem a decision maker faces when having to decide whether or not to forgo an immediate reward and wait for a delayed but potentially more substantial reward (Dai et al., 2018; Kable, 2014; Scherbaum et al., 2013). We speculate that the proposed modelling approach specific to the exploration-exploitation dilemma will enable progress into determining the computations of how the brain resolves these and other meta-control dilemmas. The key conceptual idea is to build on the assumption that control dilemmas can be formulated as an inference problem over external states (contexts), internal states (meta-control states), and control signals (actions). For example, the selection-monitoring dilemma also can be understood as a hierarchical inference problem in which an agent has to decide to which aspect of the environment it should pay attention to. The probabilistic hierarchical inference would, as we have shown here, enable an agent to infer and predict that the context might change and at the same time infer its behavioural mode, which is the most appropriate for the expected context change. One of the consequences of this inference will be that the agent will use the preferred policies for this new context and, for example, infer that different states will become task-relevant, i.e., an experimenter would measure the redirection of attention to different task features.

## Methods

### Likelihoods and transition probabilities

The latent state of the selected option is directly observable, hence the corresponding observation likelihood $p\left({l}_t|{l}_t^{\prime}\right)$ corresponds to the identity matrix. We express the relation between latent states ${s}_t^{\prime }$ and observations *o*_*t*_ as

$$ p\left({o}_t|{s}_t^{\prime },{A}^{\prime },{o}_{t-1}\right)=p\left({w}_t|{f}_t,{w}_{t-1}\right)p\left({f}_t|{l}_t^{\prime },{c}_k^{\prime },{A}^{\prime}\right)p\left({l}_t|{l}_t^{\prime}\right) $$

where the likelihood over point types *f*_*t*_ corresponds to a categorical distribution parametrised by point type probabilities *A*_*l*, *c*, *j*_

$$ p\left({f}_t|{l}_t^{\prime }=l,{c}_t^{\prime }=c,{A}^{\prime}\right)=\prod \limits_j^3{A}_{l,c,j}^{\delta_{j,{h}_t}};\kern1em {\sum}_j{A}_{l,c,j}=1 $$

where *h*_*t*_ = *h*(*f*_*t*_), which maps the point type vector *f*_*t*_ into a scalar ((0, 0) -> 1, (1, 0) -> 2, (0, 1) -> 3).

Note that the point type probabilities *A*_*l*, *c*, *j*_ has a prior set to a Dirichlet distribution

$$ p\left({A}^{\prime}\right)=\prod \limits_{l,c} Dir\left({A}_{l,c}^{\prime }|{\boldsymbol{a}}_{l,c}\right) $$

The prior parameters ***a***_*l*, *c*_ are set to form vague priors about true state-outcome contingencies. This is required to allow agent a possibility to differentiate between different contexts before any choice outcomes are observed in any context.

At the first level of the hierarchy policies *π*^′^ correspond to a sequence of five option choices, hence *π*^′^ = (*a*_1_, …, *a*_*T*_). Each choice deterministically sets the state of selected option ${l}_t^{\prime }$, thus

$$ p\left({l}_{t+1}^{\prime }|{l}_t^{\prime },{\pi}_k^{\prime}\right)=p\left({l}_{t+1}^{\prime }|{l}_t^{\prime },{a}_t\right) $$

where

$$ p\left({l}_{t+1}^{\prime }=l|{l}_t^{\prime },{a}_t\right)=\left\{\begin{array}{c}1,\kern0.5em \mathrm{if}\ {a}_t=l\\ {}\kern-1em 0,\mathrm{if}\ {a}_t\ne l\end{array}\right. $$

The auxiliary latent factors at the lower ${c}_k^{\prime },{i}_k^{\prime }$ are related to their upper level counterparts via the link probability as

$$ p\left({\pi}^{\prime },{s}_1^{\prime }|{s}_k^{\prime \prime}\right)=p\left({\pi}^{\prime }|{i}_k^{\prime}\right)p\left({i}_k^{\prime }|{i}_k^{\prime \prime}\right)p\left({c}_k^{\prime }|{c}_k^{\prime \prime}\right) $$

where ${s}_1^{\prime }=\left({l}_1^{\prime },{c}_1^{\prime },{i}_1^{\prime}\right)\equiv \left({l}_1^{\prime },{i}_k^{\prime },{c}_k^{\prime}\right)$, $p\left({i}_k^{\prime}\right|{i}_k^{\prime \prime}\Big)={I}_2,$ and $p\left({c}_k^{\prime }|{c}_k^{\prime \prime}\right)={I}_6$. Hence we define deterministic mapping between auxiliary lower level states and their upper level counterparts using identity matrices. Note that in contrast to the selected option ${l}_t^{\prime }$, latent factors ${c}_k^{\prime }$, and ${i}_k^{\prime }$ are stable during one segment. Hence, their transition probabilities correspond to identity matrix and can be ignored.

At the upper level of the hierarchy, we define the state transition probability of contexts ${c}_k^{\prime \prime }$ and context duration ${d}_k^{\prime \prime }$ in the form of an explicit duration hidden Markov model, where

$$ p\left({d}_{k+1}^{\prime \prime }=d|{d}_k^{\prime \prime}\right)=\left\{\begin{array}{c}{\delta}_{d,{d}_k^{\prime \prime }-1},\mathrm{if}{d}_k^{\prime \prime }>1\\ {}{p}_0(d),\mathrm{if}\ {d}_k^{\prime \prime }=1\end{array},\operatorname{}\right) $$

Similarly,

$$ p\left({c}_{k+1}^{\prime \prime }=c|{c}_k^{\prime \prime }=h,{d}_k^{\prime \prime}\right)=\left\{\begin{array}{c}{\delta}_{c,h},\mathrm{if}\ {d}_k^{\prime \prime }>1\\ {}\frac{1}{5}\left(1-{\delta}_{c,h}\right),\mathrm{if}\ {d}_k^{\prime \prime }=1\end{array},\operatorname{}\right) $$

where we use *J*_6_ to denote a six-dimensional all-ones matrix, and *I*_6_ a six-dimensional identity matrix. Intuitively, these state transition probabilities describe a deterministic count-down process. As long as the context duration ${d}_k^{\prime \prime }$ is above one, the context remains fixed (${c}_{k+1}^{\prime \prime }={c}_k^{\prime \prime }$) and the state duration is reduced by one (${d}_{k+1}^{\prime \prime }={d}_k^{\prime \prime }-1$). Once the duration of one is reached a new context will be uniformly selected in the next segment from the remaining five contexts, and a new context duration is sampled from the duration prior *p*_0_(*d*).

**Fig. 11:**
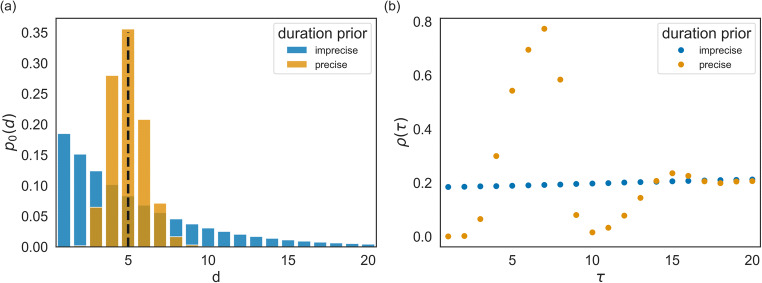
Two specific cases of duration priors and the context change predictions. (**a**) Visualisation of the precise and imprecise prior distributions of duration *d*. The dashed vertical line marks the mean of both distributions. (**b**) Effective context change probability at a future segment *k* + *τ*. The effective change probability corresponds to the probability of context change after *τ* segments conditioned on a last change in *k*th segment. Note that for precise duration prior the temporal profile of the transition probability has clearly defined periods of low and high transition probability. In the case of imprecise duration prior the change probability *ρ* is constant, corresponding to the hidden Markov model.

We will express here the duration prior as a discrete gamma distribution with bounded support, hence

$$ {p}_0(d)=\frac{1}{C}{d}^{\theta -1}{e}^{-\beta d};\kern0.75em C=\sum \limits_{d=1}^D{d}^{\theta -1}{e}^{-\beta d} $$

where D = 20. In Fig. 11a, we illustrate the duration priors for agents with precise (*θ* = 20, *β* = 4) and imprecise (*θ* = 1, *β* = 0.2) prior beliefs about the moment of change. Both priors, have the same mean but different variances. Importantly, the imprecise and precise priors correspond to imprecise and precise predictions about the future moment of change as illustrated in Fig. 11b using an effective change probability defined as

$$ \rho \left(\tau \right)=1-\sum \limits_dp\left({c}_{k+\tau}^{\prime \prime }=c|{c}_k^{\prime \prime }=c,d\right){p}_0(d),\mathrm{for}\forall c\in \left\{1,\dots, 6\right\}. $$

In other words, the effective change probability measures the probability that the current context *c* will change at some future segment *τ*. Note that the imprecise priors correspond to the standard hidden Markov model as the effective change probability remains constant.

Finally, we define the likelihood at the upper level of the hierarchy as

$$ p\left({o}_k|{i}_k^{\prime \prime }=i,{c}_k^{\prime \prime }=c,{A}^{\prime \prime}\right)=\prod \limits_{j=1}^2{A^{\prime \prime}}_{i,c,j}^{\delta_{j,{o}_k}};\kern1em \sum \limits_j{A}_{i,c,j}^{\prime \prime }=1, $$

where *o*_*k*_ = 0 if a failure at the end of the segment is observed, and *o*_*k*_ = 1 if a success is observed. Similar to the prior over likelihoods at the lower level, the prior over likelihoods at the upper level corresponds to a Dirichlet distribution

$$ p\left({A}^{\prime \prime}\right)=\prod \limits_{i,c} Dir\left({A}_{i,c}^{\prime \prime }|{\boldsymbol{b}}_{i,c}\right) $$

where ***b***_*i*, *c*_ = (1, 1) independent of the control state *i* and context *c*.

### Priors over policies – expected free energy

For our case of a hierarchical generative model, we will adapt the above relation for the expected free energy, shown in Eq. (1), and define the following priors over policies and the corresponding expected free energy at different levels of the hierarchy. Note that the epistemic and intrinsic value term are computed at both levels of the hierarchy, with the difference that the beliefs about hidden states $Q\left({s}_k^{\prime \prime}\right)$ at the second level of the hierarchy can modulate the prior preferences over policies at the lower level of the hierarchy; effectively supressing directed exploration. At the second level of the hierarchy the prior over behavioural policies, that is, the expected free energy is defined as

$$ {\displaystyle \begin{array}{rl}p\left({\pi}^{\prime \prime}\right)& =\sigma \left(-\gamma G\left({\pi}^{\prime \prime}\right)\right)\propto {e}^{-\gamma G\left({\pi}^{\prime \prime}\right)}\\ {}G\left({\pi}^{\prime \prime}\right)& ={E}_{\overset{\sim }{Q}}\left[-\ln\ Q\frac{\left({A}^{\prime \prime }|{s}_k^{\prime \prime },{o}_k,{\pi}^{\prime \prime}\right)}{Q\left({A}^{\prime \prime}\right)}-\ln\ \frac{Q\left({s}_k^{\prime \prime }|{o}_k,{\pi}^{\prime \prime}\right)}{Q\left({s}_k^{\prime \prime }|{\pi}^{\prime \prime}\right)}-U\left({o}_k\right)\right]\end{array}} $$

where *γ* = 8 , and the prior preferences over outcomes are defined as

$$ P\left({o}_k\right)\propto {e}^{U\left({o}_k\right)};\kern1em U\left({o}_k\right)=\left\{\begin{array}{c}2,\mathrm{if}\ {o}_k=\mathrm{success}\\ {}-2,\mathrm{if}\ {o}_k=\mathrm{failure}\end{array},\operatorname{}\right) $$

Importantly, as the expected free energy depends only on a single future step (segment) there are only two possible behavioural policies *π*^′′^ at the second level of the hierarchy, which sets the agent either to the first or the second control state, hence *π*^′′^ ∈ {1, 2}.

Similarly, at the first level of the hierarchy we define the expected free energy and the corresponding policy prior as

$$ {\displaystyle \begin{array}{c}p\left({\pi}^{\prime }|{i}_k^{\prime}\right)=\sigma \left(-G\left({\pi}^{\prime }|{i}_k^{\prime}\right)\right)\\ {}G\left({\pi}^{\prime }|{i}_k^{\prime}\right)=\sum \limits_{\tau =t+1}^TG\left({\pi}^{\prime },\tau |{i}_k^{\prime}\right)\\ {}G\left({\pi}^{\prime },\tau |{i}_k^{\prime}\right)=-\gamma \left({i}_k^{\prime}\right) EV\left({\pi}^{\prime },\tau \right)-\gamma \left({i}_k^{\prime}\right)\lambda \left({i}_k^{\prime}\right) IV\left({\pi}^{\prime },\tau \right)\\ {}=-\gamma \alpha \left({i}_k^{\prime}\right) EV\left({\pi}^{\prime },\tau \right)-\gamma IV\left({\pi}^{\prime },\tau \right)\\ {} EV\left({\pi}^{\prime },\tau \right)={E}_{\overset{\sim }{Q}}\left[U\left({o}_{\tau}\right)\right]\\ {} IV\left({\pi}^{\prime },\tau \right)={E}_{\overset{\sim }{Q}}\left[\ln\ \frac{Q\left(A|{s}_{\tau },{o}_{\tau },\pi \right)}{Q(A)}+\ln\ \frac{Q\left({s}_{\tau }|{o}_{\tau },\pi \right)}{Q\left({s}_{\tau }|\pi \right)}\right]\end{array}} $$

where $\gamma \left({i}_k^{\prime}\right)\lambda \left({i}_k^{\prime}\right)=\gamma =8$, and $\alpha \left({i}_k^{\prime}\right)=\frac{1}{\lambda \left({i}_k^{\prime}\right)}$. We used $\alpha \left({i}_k^{\prime}\right)$ to denote meta-control state dependent weighting of the epistemic value term in the expected free energy on the lower level. Hence, $\alpha \left({i}_k^{\prime}\right)$ controls the contribution of the epistemic value to policy selection via the auxiliary meta-control state, and consequently the second level meta-control state ${i}_k^{\prime \prime }$(as we have deterministic mapping between, e.i. $p\left({i}_k^{\prime }|{i}_k^{\prime \prime}\right)={I}_2$). By setting $\alpha \left({i}_k^{\prime}\right)=\alpha =1$, we obtain the EFE agent variant, and by setting $\alpha \left({i}_k^{\prime}\right)=\alpha =0$, we obtain the IV agent variant. These two agents are nonadaptive; hence, they have only one available meta-control state. In contrast, the adaptive agent contains two meta-control states: ${i}_k^{\prime },{i}_k^{\prime \prime}\in \left\{1,2\right\}$ states, and the weighting function,

$$ \alpha \left({i}_k^{\prime}\right)=\left\{\begin{array}{c}1,\kern0.5em \mathrm{for}\ {i}_k^{\prime \prime }=1\\ {}0,\kern0.5em \mathrm{for}\ {i}_k^{\prime \prime }=2\end{array}\right. $$

Finally, we defined the outcome utility at the first level of the hierarchy as

$$ U\left({o}_{\tau}\right)=U\left({w}_{\tau}\right)=\left\{\begin{array}{c}1,\mathrm{if}\ {w}_{\tau}^{blue}\ge 4\  or\ {w}_{\tau}^{red}\ge 4,\mathrm{and}\ \tau =T\\ {}0,\mathrm{otherwise}\end{array}\right. $$

The behavioural policies at the first level of the hierarchy correspond to a set of sequences of all possible choices (option selection). Hence, *π*^′^ ∈ {1, …, 1024}.

### Variational inference

Inverting the generative model requires computing posterior beliefs over hidden states and behavioural policies at different levels of the hierarchy. This computation is analytically intractable and can be approximated using variational inference. Under the mean-field approximation, the true posterior is approximated as a product of multiple independent factors, hence

$$ p\left({A}^{\prime \prime },{\pi}^{\prime \prime },{s}_k^{\prime \prime },{A}^{\prime },{\pi}^{\prime },{s}_{1:T}^{\prime }|{\left[{O}_T^{\prime}\right]}^{1:k},{O}_k^{\prime \prime}\right)\approx Q\left({A}^{\prime \prime}\right)Q\left({\pi}^{\prime \prime}\right)Q\left({s}_k^{\prime \prime }|{\pi}^{\prime \prime}\right)Q\left({A}^{\prime}\right)\ Q\left({\pi}^{\prime}\right)Q\left({S}_T^{\prime }|{\pi}^{\prime}\right) $$

where $$ {\left[{O}_T^{\prime}\right]}^{1:k}=\left({o}_1^1,\dots, {o}_T^1,\dots, {o}_1^k,\dots, {o}_T^k\right) $$, $$ {O}_k^{\prime \prime }=\left({o}_1,\dots, {o}_k\right) $$, $$ {S}_T^{\prime }=\left({s}_1^{\prime },\dots, {s}_T^{\prime}\right) $$.

The approximate posterior is found as the minimiser of the variational free energy

$$ F=\int dxQ(x)\ln \frac{Q(x)}{p\left({o}_k,{o}_{1:T}^k|x\right)\overline{p}(x)} $$

where $x=\left({A}^{\prime \prime },{\pi}^{\prime \prime },{s}_k^{\prime \prime },{A}^{\prime },{\pi}^{\prime },{S}_T^{\prime}\right),\mathrm{and}\ \overline{p}(x)=p\left(x|{\left[{O}_T^{\prime}\right]}^{1:k-1},{O}_{k-1}^{\prime \prime}\right).$ The minimum of the variational free energy corresponds to the following relations:

- *Upper level*
  $$ {\displaystyle \begin{array}{l}Q\left(A^{{\prime\prime}}\right)\propto \overline{p}\left(A^{{\prime\prime}}\right)\exp \left\{\sum \limits_{s_k^{\prime }}Q\left({s}_k^{{\prime\prime}}\right)\ln p\left({o}_k|{s}_k^{{\prime\prime} },A^{{\prime\prime}}\right)\right\}\\ {}Q\left(\pi^{{\prime\prime}}\right)\propto p\left(\pi^{{\prime\prime}}\right)\exp \left\{-\sum \limits_{s_k^{\prime }}Q\left({s}_k^{{\prime\prime} }|\pi^{{\prime\prime}}\right)\ln \frac{Q\left({s}_k^{{\prime\prime} }|\pi^{{\prime\prime}}\right)}{\overline{p}\left({o}_k,{s}_k^{{\prime\prime} }|\pi^{{\prime\prime}}\right)}\right\}\\ {}Q\left({s}_k^{{\prime\prime} }|\pi^{{\prime\prime}}\right)\propto \overline{p}\left({o}_k|{s}_k^{{\prime\prime}}\right)\overline{p}\left({s}_k^{{\prime\prime} }|\pi^{{\prime\prime}}\right)\exp \left\{\sum \limits_{c_k^{\prime },{i}_k^{\prime }}Q\left({i}_k^{\prime}\right)Q\left({c}_k^{\prime}\right)\ln p\left({i}_k^{\prime },{c}_k^{\prime }|{s}_k^{{\prime\prime}}\right)\right\}\end{array}} $$

where

$$ \overline{p}\left({o}_k|{s}_k^{{\prime\prime}}\right)=\int d{A}^{{\prime\prime}}\overline{p}\left({A}^{{\prime\prime}}\right)p\left({o}_k|{s}_k,{A}^{{\prime\prime}}\right), $$

$$ \overline{p}\left({s}_k^{{\prime\prime} }|{\pi}^{{\prime\prime}}\right)={\sum}_{s_{k-1}^{{\prime\prime} }}p\left({s}_k^{{\prime\prime} }|{s}_{k-1}^{{\prime\prime} },{\pi}^{{\prime\prime}}\right)Q\left({s}_{k-1}^{{\prime\prime}}\right). $$

- *Lower level*
  $$ {\displaystyle \begin{array}{r}Q\left({\pi}^{\prime}\right)\propto \exp\ \left\{{\sum}_{s_k^{\prime }}Q\left({i}_k^{\prime}\right)\ln\ p\Big({\pi}^{\prime }|{i}_k^{\prime}\Big)-F\left({\pi}^{\prime}\right)\right\}=\exp\ \left\{-\gamma \left[{\overline{\alpha}}_k EV\left({\pi}^{\prime },\tau \right)- IV\left({\pi}^{\prime },\tau \right)\right]-F\left({\pi}^{\prime}\right)\right\}\\ {}F\left({\pi}^{\prime}\right)={E}_{Q\left({A}^{\prime}\right)}\left[{\sum}_{l_{1:t}^{\prime },{s}_k^{\prime }}Q\left({c}_k^{\prime}\right)Q\left(\ {S}_t^{\prime }|{\pi}^{\prime}\right)\ln\ \frac{Q\left({c}_k^{\prime}\right)Q\left({S}_t^{\prime }|{\pi}^{\prime}\right)}{\overset{\sim }{p}\left({\left[{O}_t^{\prime}\right]}^k|{S}_t^{\prime },{c}_k^{\prime },{A}^{\prime}\right)p\left({S}_t^{\prime }|{\pi}^{\prime}\right)\overset{\sim }{p}\left({c}_k^{\prime}\right)}\right]\end{array}} $$

where $\overset{\sim }{p}\Big({\left[{O}_t^{\prime}\right]}^k\left|{S}_t^{\prime },{A}^{\prime}\right)={\prod}_{j=1}^tp\left({o}_j^k|{l}_j^{\prime },{c}_k^{\prime },{A}^{\prime}\right)$, $\overset{\sim }{p}\left({i}_k^{\prime}\right)={\sum}_{i_k^{\prime \prime }}Q\left({i}_k^{\prime \prime}\right)p\left({i}_k^{\prime }|{i}_k^{\prime \prime}\right),$and $\overset{\sim }{p}\left({c}_k^{\prime}\right)={\sum}_{c_k^{\prime \prime }}Q\left({c}_k^{\prime \prime}\right)p\left({c}_k^{\prime }|{c}_k^{\prime \prime}\right)$. To estimate the beliefs over a sequence of locations ${l}_{1:t}^{\prime }$, and a fixed context${c}_k^{\prime }$, we use the Bethe approximation and the corresponding belief propagation algorithm (Schwöbel, Kiebel and Marković, 2018)

$$ {\displaystyle \begin{array}{c}Q\left({l}_t^{\prime }|{\pi}^{\prime}\right)\propto \exp \left\{\mathit{\ln}\overline{p}\left({o}_t\right|{l}_t^{\prime}\right)+\ln \overrightarrow{m}\left({l}_t^{\prime }|{\pi}^{\prime}\right)+\ln \overleftarrow{m}\left({l}_t^{\prime }|{\pi}^{\prime}\right)\Big\}\\ {}\mathrm{Q}\left({c}_{\mathrm{k}}^{\prime}\right)\propto \overset{\sim }{p}\left({c}_k^{\prime}\right)\exp \left\{\ln \overline{p}\left({\left[{O}_t^{\prime}\right]}^k|{c}_k^{\prime}\right)\right\}\\ {}\begin{array}{c}Q\left({i}_k^{\prime}\right)\propto \overset{\sim }{p}\left({i}_k^{\prime}\right)\exp \left\{\sum \limits_{\pi^{\prime }}Q\left({\pi}^{\prime}\right)\mathit{\ln}p\left({\pi}^{\prime }|{i}_k^{\prime}\right)\right\}\\ {}\ln \overline{p}\left({o}_t|{l}_t^{\prime}\right)=\sum \limits_{c_k^{\prime }}Q\left({c}_k^{\prime}\right)\int d{A}^{\prime }Q\left({A}^{\prime}\right)\ln p\left({o}_t|{l}_t^{\prime },{c}_k^{\prime },{A}^{\prime}\right)\kern0.5em \\ {}\ln \overline{p}\left({\left[{O}_t^{\prime}\right]}^k|{c}_k^{\prime}\right)=\sum \limits_{l_{1:t}^{\prime },{\pi}^{\prime }}Q\left({l}_{1:t}^{\prime }|{\pi}^{\prime}\right)Q\left({\pi}^{\prime}\right)\int d{A}^{\prime }Q\left({A}^{\prime}\right)\sum \limits_{j=1}^t\ln p\left({o}_j^k|{l}_j^{\prime },{c}_k^{\prime },{A}^{\prime}\right)\end{array}\end{array}} $$

Finally, we obtain the posterior beliefs over likelihood point type probabilities at the first level of the hierarchy likelihoods as

$$ Q\left({A}^{\prime}\right)\propto \overline{\mathrm{p}}\left({\mathrm{A}}^{\prime}\right)\exp \left\{\sum \limits_{t=1}^T\sum \limits_{l_t^{\prime },{c}_k^{\prime }}Q\left({l}_t^{\prime }|{\pi}^{\prime}\right)Q\left({c}_k^{\prime}\right)Q\left({\pi}^{\prime}\right)\ln p\left({o}_t\right|{l}_t^{\prime },{c}_k^{\prime },{\mathrm{A}}^{\prime}\Big)\right\} $$

Note that we used a product of Dirichlet distributions as the prior and the posterior over likelihoods at the two levels of the hierarchy; hence, we write

$$ {\displaystyle \begin{array}{rl}\overline{p}\left({A}^{\prime}\right)& =\prod \limits_{c,l} Dir\left({\boldsymbol{a}}_{c,l}^{k-1}\right)\\ {}\overline{p}\left({A}^{\prime \prime}\right)& =\prod \limits_{c,i} Dir\left({\boldsymbol{b}}_{c,i}^{k-1}\right)\end{array}} $$

and the corresponding approximate posterior as

$$ {\displaystyle \begin{array}{rl}Q\left({A}^{\prime}\right)& =\prod \limits_{c,l} Dir\left({\boldsymbol{a}}_{c,l}^k\right)\\ {}Q\left({A}^{\prime \prime}\right)& =\prod \limits_{c,i} Dir\left({\boldsymbol{b}}_{c,i}^k\right)\end{array}} $$

Thus, the update equations for the parameters of the Dirichlet posterior become

$$ {\displaystyle \begin{array}{rl}{a}_{c,l,j}^k& ={a}_{c,l,j}^{k-1}+{\delta}_{j,{h}_t}\cdot Q\left({l}_t^{\prime }=l\right)Q\left({c}_k^{\prime }=c\right)\\ {}{b}_{c,i,j}^k& ={b}_{c,i,j}^{k-1}+{\delta}_{j,{o}_k}\cdot Q\left({i}_t^{\prime \prime }=i\right)Q\left({c}_k^{\prime \prime }=c\right)\end{array}} $$

### Statistics

We use the following definitions of the group mean success rate and success probability. Let ${O}_{K,n}^{\prime \prime }$ be the sequence of outcomes (successes – 1, failures 0) at the second level of the hierarchy for the *n*th simulation after *K* = 200 segments. Then the group mean success rate at *k*th segment is defined as

$$ {\left\langle {o}_k\right\rangle}_{group}=\frac{1}{N}\sum \limits_{n=1}^N{\left[{o}_k\right]}_n $$

Similarly, to define the instance-specific success probability, we use the following relation

$$ \left\langle {O}_{K,n}^{\prime \prime}\right\rangle =\frac{1}{M}\sum \limits_{k\in \Omega}{\left[{o}_k\right]}_n $$

where Ω denotes set of valid segments, and *M* = ∣ Ω∣. For example, when computing success probability at different time points (relative segment numbers) of a repeated context type, the set of valid segment Ω will consist of a sequence (101, 106, …) for the relative segment number *r* = 0, of a sequence (102, 107, …), for the relative segment number *r* = 1,and so on for the three remaining relative segment numbers.

## References

[CR1] Addicott MA, Pearson JM, Sweitzer MM, Barack DL, Platt ML (2017). A primer on foraging and the explore/exploit trade-off for psychiatry research. Neuropsychopharmacology.

[CR2] Agrawal, S., & Goyal, N. (2012). *Analysis of thompson sampling for the multi-armed bandit problem.* Paper presented at the Conference on learning theory.

[CR3] Allesiardo R, Féraud R, Maillard O-A (2017). The non-stationary stochastic multi-armed bandit problem. International Journal of Data Science and Analytics.

[CR4] Auer P, Cesa-Bianchi N, Fischer P (2002). Finite-time analysis of the multiarmed bandit problem. Machine Learning.

[CR5] Bacon, P.-L., Harb, J., & Precup, D. (2017). *The option-critic architecture.* Paper presented at the Thirty-First AAAI Conference on Artificial Intelligence.

[CR6] Bacon PL, Precup D (2018). Constructing Temporal Abstractions Autonomously in Reinforcement Learning. AI Magazine.

[CR7] Badre D, Nee DE (2018). Frontal Cortex and the Hierarchical Control of Behavior. Trends in Cognitive Sciences.

[CR8] Barto AG, Mahadevan S (2003). Recent advances in hierarchical reinforcement learning. Discrete Event Dynamic Systems.

[CR9] Behrens TE, Woolrich MW, Walton ME, Rushworth MF (2007). Learning the value of information in an uncertain world. Nature Neuroscience.

[CR10] Blanchard TC, Gershman SJ (2018). Pure correlates of exploration and exploitation in the human brain. Cognitive, Affective, & Behavioral Neuroscience.

[CR11] Botvinick M, Toussaint M (2012). Planning as inference. Trends in Cognitive Sciences.

[CR12] Botvinick M, Weinstein A (2014). Model-based hierarchical reinforcement learning and human action control. Philosophical Transactions of the Royal Society, B: Biological Sciences.

[CR13] Botvinick MM, Cohen JD (2014). The Computational and Neural Basis of Cognitive Control: Charted Territory and New Frontiers. Cognitive Science.

[CR14] Botvinick MM, Niv Y, Barto AG (2009). Hierarchically organized behavior and its neural foundations: A reinforcement learning perspective. Cognition.

[CR15] Boureau Y-L, Sokol-Hessner P, Daw ND (2015). Deciding how to decide: Self-control and meta-decision making. Trends in Cognitive Sciences.

[CR16] Chaudhuri R, Knoblauch K, Gariel MA, Kennedy H, Wang XJ (2015). A Large-Scale Circuit Mechanism for Hierarchical Dynamical Processing in the Primate Cortex. Neuron.

[CR17] Cohen, J. D. (2017). Core Constructs and Current Considerations. In T. Egner (Ed.), *The Wiley Handbook of Cognitive Control*: Wiley-Blackwell.

[CR18] Cohen JD, McClure SM, Yu AJ (2007). Should I stay or should I go? How the human brain manages the trade-off between exploitation and exploration. Philosophical Transactions of the Royal Society, B: Biological Sciences.

[CR19] Collin SHP, Milivojevic B, Doeller CF (2017). Hippocampal hierarchical networks for space, time, and memory. Current Opinion in Behavioral Sciences.

[CR20] Collins, A., & Koechlin, E. (2012). Reasoning, Learning, and Creativity: Frontal Lobe Function and Human Decision-Making. *PLoS Biology, 10*(3). 10.1371/journal.pbio.1001293 10.1371/journal.pbio.1001293 PMC3313946 22479152

[CR21] Cuevas Rivera D, Ott F, Marković D, Strobel A, Kiebel SJ (2018). Context-dependent risk aversion: a model-based approach. Frontiers in Psychology.

[CR22] Dai JY, Pleskac TJ, Pachur T (2018). Dynamic cognitive models of intertemporal choice. Cognitive Psychology.

[CR23] Daw ND, Doya K (2006). The computational neurobiology of learning and reward. Current Opinion in Neurobiology.

[CR24] Daw ND, O'Doherty JP, Dayan P, Seymour B, Dolan RJ (2006). Cortical substrates for exploratory decisions in humans. Nature.

[CR25] Dayan, P., & Angela, J. Y. (2003). *Expected and unexpected uncertainty: ACh and NE in the neocortex.* Paper presented at the Advances in neural information processing systems.

[CR26] Dezza IC, Angela JY, Cleeremans A, Alexander W (2017). Learning the value of information and reward over time when solving exploration-exploitation problems. Scientific Reports.

[CR27] Dixon, M. L., Girn, M., & Christoff, K. (2017). Hierarchical Organization of Frontoparietal Control Networks Underlying Goal-Directed Behavior. In: M. Watanabe (Ed.), *The Prefrontal Cortex as an Executive, Emotional, and Social Brain*: Springer.

[CR28] Doya K (2002). Metalearning and neuromodulation. Neural Networks.

[CR29] Dreisbach G, Goschke T (2004). How positive affect modulates cognitive control: Reduced perseveration at the cost of increased distractibility. Journal of Experimental Psychology-Learning Memory and Cognition.

[CR30] Dubins, L. E., Savage, L. J., Sudderth, W., & Gilat, D. (2014). *How to gamble if you must: Inequalities for stochastic processes*: Courier Corporation.

[CR31] Duverne, S., & Koechlin, E. (2017). Hierarchical Control of Behaviour in Human Prefrontal Cortex. In T. Egner (Ed.), *The Wiley Handbook of Cognitive Control*: John Wiley & Sons Ltd.

[CR32] Economides M, Guitart-Masip M, Kurth-Nelson Z, Dolan RJ (2014). Anterior Cingulate Cortex Instigates Adaptive Switches in Choice by Integrating Immediate and Delayed Components of Value in Ventromedial Prefrontal Cortex. Journal of Neuroscience.

[CR33] Egner, T. (2017). Conflict Adaptation: Past, Present, and Future of the Congruency Sequence Effect as an Index of Cognitive Control. In T. Egner (Ed.), *The Wiley Handbook of Cognitive Control*: Wiley-Blackwell.

[CR34] FitzGerald TH, Hämmerer D, Friston KJ, Li S-C, Dolan RJ (2017). Sequential inference as a mode of cognition and its correlates in fronto-parietal and hippocampal brain regions. PLoS Computational Biology.

[CR35] FitzGerald TH, Schwartenbeck P, Moutoussis M, Dolan RJ, Friston K (2015). Active inference, evidence accumulation, and the urn task. Neural Computation.

[CR36] Friston K (2010). The free-energy principle: a unified brain theory?. Nature Reviews Neuroscience.

[CR37] Friston K, Rigoli F, Ognibene D, Mathys C, Fitzgerald T, Pezzulo G (2015). Active inference and epistemic value. Cognitive Neuroscience.

[CR38] Friston KJ, Rosch R, Parr T, Price C, Bowman H (2018). Deep temporal models and active inference. Neuroscience & Biobehavioral Reviews.

[CR39] Garbusow M, Schad DJ, Sommer C, Junger E, Sebold M, Friedel E (2014). Pavlovian-to-Instrumental Transfer in Alcohol Dependence: A Pilot Study. Neuropsychobiology.

[CR40] Garivier, A., & Cappé, O. (2011). *The KL-UCB algorithm for bounded stochastic bandits and beyond.* Paper presented at the Proceedings of the 24th annual conference on learning theory.

[CR41] Geana, A., Wilson, R., Daw, N. D., & Cohen, J. D. (2016). *Boredom, Information-Seeking and Exploration.* Paper presented at the CogSci.

[CR42] Gershman SJ, Horvitz EJ, Tenenbaum JB (2015). Computational rationality: A converging paradigm for intelligence in brains, minds, and machines. Science.

[CR43] Ghavamzadeh MM, Pineau J, Tamar A (2015). Bayesian Reinforcement Learning: A Survey. Foundations and Trends R in Machine Learning.

[CR44] Gollwitzer PM, Bargh JA (1996). *The psychology of action: Linking cognition and motivation to behavior*.

[CR45] Goschke T, Roth SMWPG (2003). Voluntary action and cognitive control from a cognitive neuroscience perspective. Voluntary action: Brains, minds, and sociality. *Voluntary action: Brains, minds, and sociality*.

[CR46] Goschke T, Herwig WPABA (2013). Volition in action: Intentions, control dilemmas and the dynamic regulation of intentional control. *Action science: Foundations of an emerging discipline*.

[CR47] Goschke T, Bolte A (2014). Emotional modulation of control dilemmas: The role of positive affect, reward, and dopamine in cognitive stability and flexibility. Neuropsychologia.

[CR48] Goschke T, Dreisbach G (2008). Conflict-triggered goal shielding: Response conflicts attenuate background monitoring for prospective memory cues. Psychological Science.

[CR49] Gruber O, Diekhof EK, Kirchenbauer L, Goschke T (2010). A neural system for evaluating the behavioural relevance of salient events outside the current focus of attention. Brain Research.

[CR50] Gupta, N., Granmo, O.-C., & Agrawala, A. (2011). *Thompson sampling for dynamic multi-armed bandits.* Paper presented at the 2011 10th International Conference on Machine Learning and Applications and Workshops.

[CR51] Hasson U, Yang E, Vallines I, Heeger DJ, Rubin N (2008). A hierarchy of temporal receptive windows in human cortex. Journal of Neuroscience.

[CR52] Heckhausen H, Kuhl J, Frese M, Sabini J (1985). From wishes to action: The dead ends and short cuts on the long way to action. *Goal directed behavior*.

[CR53] Heilbronner SR, Hayden BY (2016). Dorsal Anterior Cingulate Cortex: A Bottom-Up View. Annual Review of Neuroscience.

[CR54] Holroyd CB, McClure SM (2015). Hierarchical control over effortful behavior by rodent medial frontal cortex: A computational model. Psychological Review.

[CR55] Houthooft, R., Chen, X., Duan, Y., Schulman, J., De Turck, F., & Abbeel, P. (2016). *Vime: Variational information maximizing exploration.* Paper presented at the Advances in Neural Information Processing Systems.

[CR56] Hunt LT, Hayden BY (2017). A distributed, hierarchical and recurrent framework for reward-based choice. Nature Reviews. Neuroscience.

[CR57] Izquierdo A, Brigman JL, Radke AK, Rudebeck PH, Holmes A (2017). The neural basis of reversal learning: an updated perspective. Neuroscience.

[CR58] Kable JW, Glimcher P, Fehr E (2014). Valuation, Intertemporal Choice, and Self-Control. *Neuroeconomics (Second Edition) Decision Making and the Brain*.

[CR59] Kaelbling LP, Littman ML, Cassandra AR (1998). Planning and acting in partially observable stochastic domains. Artificial Intelligence.

[CR60] Kalanthroff E, Davelaar EJ, Henik A, Goldfarb L, Usher M (2018). Task Conflict and Proactive Control: A Computational Theory of the Stroop Task. Psychological Review.

[CR61] Kaplan R, Friston KJ (2018). Planning and navigation as active inference. Biological Cybernetics.

[CR62] Kiebel SJ, Daunizeau J, Friston KJ (2008). A hierarchy of time-scales and the brain. PLoS Computational Biology.

[CR63] Koch I, Poljac E, Muller H, Kiesel A (2018). Cognitive Structure, Flexibility, and Plasticity in Human Multitasking-An Integrative Review of Dual-Task and Task-Switching Research. Psychological Bulletin.

[CR64] Koechlin E, Ody C, Kouneiher F (2003). The architecture of cognitive control in the human prefrontal cortex. Science.

[CR65] Kolling N, Behrens TEJ, Mars RB, Rushworth MFS (2012). Neural Mechanisms of Foraging. Science.

[CR66] Kolling N, Wittmann M, Rushworth MFS (2014). Multiple neural mechanisms of decision making and their competition under changing risk pressure. Neuron.

[CR67] Kuhl J, Goschke T, Kuhl J, Beckmann J (1994). A theory of action control: Mental subsystems, modes of control, and volitional conflict-resolution strategies. *Volition and personality: Action versus state orientation*.

[CR68] Kurniawati H, Du YZ, Hsu D, Lee WS (2011). Motion planning under uncertainty for robotic tasks with long time horizons. International Journal of Robotics Research.

[CR69] Laureiro-Martínez D, Brusoni S, Canessa N, Zollo M (2015). Understanding the exploration–exploitation dilemma: An fMRI study of attention control and decision-making performance. Strategic Management Journal.

[CR70] Le TP, Vien NA, Chung T (2018). A Deep Hierarchical Reinforcement Learning Algorithm in Partially Observable Markov Decision Processes. Ieee Access.

[CR71] Lewis RL, Howes A, Singh S (2014). Computational rationality: Linking mechanism and behavior through bounded utility maximization. Topics in Cognitive Science.

[CR72] Lieder F, Griffiths TL (2017). Strategy selection as rational metareasoning. Psychological Review.

[CR73] Littman ML (2009). A tutorial on partially observable Markov decision processes. Journal of Mathematical Psychology.

[CR74] Liu H, Liu K, Zhao Q (2012). Learning in a changing world: Restless multiarmed bandit with unknown dynamics. IEEE Transactions on Information Theory.

[CR75] Maisto D, Friston K, Pezzulo G (2019). Caching mechanisms for habit formation in Active Inference. Neurocomputing.

[CR76] Marković D, Reiter AM, Kiebel SJ (2019). Predicting change: Approximate inference under explicit representation of temporal structure in changing environments. PLoS Computational Biology.

[CR77] Mathys C, Daunizeau J, Friston KJ, Stephan KE (2011). A Bayesian foundation for individual learning under uncertainty. Frontiers in Human Neuroscience.

[CR78] McGuire JT, Nassar MR, Gold JI, Kable JW (2014). Functionally dissociable influences on learning rate in a dynamic environment. Neuron.

[CR79] Meyniel F, Maheu M, Dehaene S (2016). Human inferences about sequences: A minimal transition probability model. PLoS Computational Biology.

[CR80] Meyniel F, Sigman M, Mainen ZF (2015). Confidence as Bayesian probability: From neural origins to behavior. Neuron.

[CR81] Miller EK, Cohen JD (2001). An integrative theory of prefrontal cortex function. Annual Review of Neuroscience.

[CR82] Miller GA, Galanter E, Pribram KH (1960). *Plans and the structure of behavior*.

[CR83] Mnih V, Kavukcuoglu K, Silver D, Rusu AA, Veness J, Bellemare MG (2015). Human-level control through deep reinforcement learning. Nature.

[CR84] Musslick, S., Jang, S. J., Shvartsman, M., Shenhav, A., & Cohen, J. D. (2018). *Constraints associated with cognitive control and the stability-flexibility dilemma.* Paper presented at the CogSci.

[CR85] Nassar MR, Wilson RC, Heasly B, Gold JI (2010). An approximately Bayesian delta-rule model explains the dynamics of belief updating in a changing environment. Journal of Neuroscience.

[CR86] Pang, Z. J., Liu, R. Z., Meng, Z. Y., Zhang, Y., Yu, Y., Lu, T., & Aaai. (2019). *On Reinforcement Learning for Full-Length Game of StarCraft*.

[CR87] Parr, T., & Friston, K. J. (2019). Generalised free energy and active inference. *Biological Cybernetics*10.1007/s00422-019-00805-w 10.1007/s00422-019-00805-w PMC6848054 31562544

[CR88] Pezzulo G, Rigoli F, Friston K (2015). Active Inference, homeostatic regulation and adaptive behavioural control. Progress in Neurobiology.

[CR89] Scherbaum S, Dshemuchadse M, Leiberg S, Goschke T (2013). Harder than Expected: Increased Conflict in Clearly Disadvantageous Delayed Choices in a Computer Game. PLoS One.

[CR90] Scherbaum S, Dshemuchadse M, Ruge H, Goschke T (2012). Dynamic goal states: Adjusting cognitive control without conflict monitoring. Neuroimage.

[CR91] Scherbaum S, Fischer R, Dshemuchadse M, Goschke T (2011). The dynamics of cognitive control: Evidence for within-trial conflict adaptation from frequency-tagged EEG. Psychophysiology.

[CR92] Schlagenhauf F, Huys QJ, Deserno L, Rapp MA, Beck A, Heinze H-J (2014). Striatal dysfunction during reversal learning in unmedicated schizophrenia patients. Neuroimage.

[CR93] Schulz E, Gershman SJ (2019). The algorithmic architecture of exploration in the human brain. Current Opinion in Neurobiology.

[CR94] Schwartenbeck P, FitzGerald TH, Mathys C, Dolan R, Friston K (2015). The Dopaminergic Midbrain Encodes the Expected Certainty about Desired Outcomes. Cerebral Cortex.

[CR95] Schwartenbeck P, Passecker J, Hauser TU, FitzGerald TH, Kronbichler M, Friston KJ (2019). Computational mechanisms of curiosity and goal-directed exploration. eLife.

[CR96] Schwarting, W., Alonso-Mora, J., & Rus, D. (2018). Planning and Decision-Making for Autonomous Vehicles. In N. E. Leonard (Ed.), *Annual Review of Control, Robotics, and Autonomous Systems, Vol 1* (Vol. 1, pp. 187-210). Palo Alto: Annual Reviews.

[CR97] Schwöbel S, Kiebel S, Marković D (2018). Active inference, belief propagation, and the bethe approximation. Neural Computation.

[CR98] Shenhav A, Botvinick MM, Cohen JD (2013). The expected value of control: an integrative theory of anterior cingulate cortex function. Neuron.

[CR99] Shenhav A, Straccia MA, Musslick S, Cohen JD, Botvinick MM (2018). Dissociable neural mechanisms track evidence accumulation for selection of attention versus action. Nature Communications.

[CR100] Silver D, Schrittwieser J, Simonyan K, Antonoglou I, Huang A, Guez A (2017). Mastering the game of Go without human knowledge. Nature.

[CR101] Soltani A, Izquierdo A (2019). Adaptive learning under expected and unexpected uncertainty. Nature Reviews Neuroscience.

[CR102] Speekenbrink M, Konstantinidis E (2015). Uncertainty and exploration in a restless bandit problem. Topics in Cognitive Science.

[CR103] Sutton RS, Precup D, Singh S (1999). Between MDPs and semi-MDPs: A framework for temporal abstraction in reinforcement learning. Artificial Intelligence.

[CR104] Wilson RC, Geana A, White JM, Ludvig EA, Cohen JD (2014). Humans use directed and random exploration to solve the explore–exploit dilemma. Journal of Experimental Psychology: General.

[CR105] Yu S-Z (2010). Hidden semi-Markov models. Artificial Intelligence.

